# Pepinemab antibody blockade of SEMA4D in early Huntington’s disease: a randomized, placebo-controlled, phase 2 trial

**DOI:** 10.1038/s41591-022-01919-8

**Published:** 2022-08-08

**Authors:** Andrew Feigin, Elizabeth E. Evans, Terrence L. Fisher, John E. Leonard, Ernest S. Smith, Alisha Reader, Vikas Mishra, Richard Manber, Kimberly A. Walters, Lisa Kowarski, David Oakes, Eric Siemers, Karl D. Kieburtz, Maurice Zauderer, the Huntington Study Group SIGNAL investigators, Elise Kayson, Jody Goldstein, Richard Barbano, Karen Marder, Praveen Dayalu, Herminia Diana Rosas, Sandra Kostyk, John Kamholz, Brad Racette, Jee Bang, Daniel Claassen, Katherine McDonell, Stewart Factor, Francis Walker, Clarisse Goas, Joanne Wojcieszek, Lynn A. Raymond, Jody Corey-Bloom, Victor Sung, Marissa Dean, Michael Geshwind, Alexandra Nelson, Samuel Frank, Kathrin LaFaver, Andrew Duker, Lawrence Elmer, Ali Samii, Yi-Han Lin, Sylvain Chouinard, Lauren Seeberger, Burton Scott, James Boyd, Nikolaus McFarland, Erin Furr Stimming, Oksana Suchowersky, Claudia Testa, Karen Anderson

**Affiliations:** 1 New York University Langone Health and The Marlene and Paolo Fresco Institute for Parkinson’s and Movement Disorders, New York, NY USA; 2 Vaccinex, Inc., Research, Rochester, NY USA; 3 IXICO, London, UK; 4 WCG Statistics Collaborative, Inc., Washington, DC USA; 5 University of Rochester Medical Center, Rochester, NY USA; 6 Siemers Integration LLC, Zionsville, IN USA; 7 Clinical Trials Coordination Center, University of Rochester, Rochester, NY USA; 8 Columbia University, New York, NY USA; 9 University of Michigan, Ann Arbor, MI USA; 10 Massachusetts General Hospital, Boston, MA USA; 11 Ohio State University, Columbus, OH USA; 12 University of Iowa, Iowa City, IA USA; 13 Washington University, St. Louis, MO USA; 14 Johns Hopkins University, Baltimore, MD USA; 15 Vanderbuilt University, Nashville, TN USA; 16 Emory University, Atlanta, GA USA; 17 Wake Forest University, Winston-Salem, NC USA; 18 Indiana University, Bloomington, IN USA; 19 University of British Columbia, Vancouver, British Columbia Canada; 20 UCSD, San Diego, CA USA; 21 University of Alabama at Birmingham, Birmingham, AL USA; 22 UCSF, San Francisco, CA USA; 23 Beth Israel Deaconess Medical Center, Boston, MA USA; 24 University of Louisville, Louisville, KY USA; 25 University of Cincinnati, Cincinnati, OH USA; 26 University of Toledo, Toledo, OH USA; 27 University of Washington, Seattle, WA USA; 28 CHUM, Montreal, Quebec Canada; 29 University of Colorado, Aurora, CO USA; 30 Duke University, Durham, NC USA; 31 University of Vermont, South Burlington, VT USA; 32 University of Florida Gainesville, Gainesville, FL USA; 33 University of Texas Houston, Houston, TX USA; 34 University of Alberta, Edmonton, Alberta Canada; 35 University of North Carolina at Chapel Hill, Chapel Hill, NC USA; 36 Georgetown University, Washington, DC USA

**Keywords:** Huntington's disease, Drug development

## Abstract

SIGNAL is a multicenter, randomized, double-blind, placebo-controlled phase 2 study (no. NCT02481674) established to evaluate pepinemab, a semaphorin 4D (SEMA4D)-blocking antibody, for treatment of Huntington’s disease (HD). The trial enrolled a total of 265 HD gene expansion carriers with either early manifest (EM, *n* = 179) or late prodromal (LP, *n* = 86) HD, randomized (1:1) to receive 18 monthly infusions of pepinemab (*n* = 91 EM, 41 LP) or placebo (*n* = 88 EM, 45 LP). Pepinemab was generally well tolerated, with a relatively low frequency of serious treatment-emergent adverse events of 5% with pepinemab compared to 9% with placebo, including both EM and LP participants. Coprimary efficacy outcome measures consisted of assessments within the EM cohort of (1) a two-item HD cognitive assessment family comprising one-touch stockings of Cambridge (OTS) and paced tapping (PTAP) and (2) clinical global impression of change (CGIC). The differences between pepinemab and placebo in mean change (95% confidence interval) from baseline at month 17 for OTS were −1.98 (−4.00, 0.05) (one-sided *P* = 0.028), and for PTAP 1.43 (−0.37, 3.23) (one-sided *P* = 0.06). Similarly, because a significant treatment effect was not observed for CGIC, the coprimary endpoint, the study did not meet its prespecified primary outcomes. Nevertheless, a number of other positive outcomes and post hoc subgroup analyses—including additional cognitive measures and volumetric magnetic resonance imaging and fluorodeoxyglucose–positron-emission tomography imaging assessments—provide rationale and direction for the design of a phase 3 study and encourage the continued development of pepinemab in patients diagnosed with EM HD.

## Abstract (web summary)

The SIGNAL Phase 2 study of pepinemab immunotherapy in early Huntington’s disease (HD) did not meet its coprimary clinical efficacy endpoints, but had a favorable safety profile and showed a significant treatment-related reduction in caudate brain atrophy and reversal of the characteristic decline in brain metabolic activity that is typical of HD progression.

## Main

Huntington’s disease is a progressive fatal neurodegenerative disease that affects between five and ten per 100,000 people of European descent, but is less prevalent in Asian and African countries. More than 30,000 Americans are diagnosed with manifest disease and approximately another 150,000 are at risk of having inherited the dominant mutation, with approximately equal prevalence in Europe and Australia. To date, no potential disease-modifying therapy has demonstrated efficacy. Currently available treatments focus on managing psychiatric and motor symptoms such as chorea, but may be limited by poor tolerability and short duration and they may not produce meaningful functional benefits. Patients and caregivers have identified cognitive and emotional impairment as the most substantial issues impacting their lives^1,2^. Neuropathologic changes associated with toxic aggregates of mutant huntingtin protein are characterized by brain atrophy, neuronal loss and dysfunction. The most pronounced changes occur in striatal medium spiny neurons but spread to other brain regions (for example, cortex and white matter)^3^. Evidence from imaging studies in patients with HD and animal models suggests that neuronal dysfunction is accompanied by major changes in glial cells, including loss of normal functions and gain of reactive neurotoxic and neuroinflammatory responses. These changes occur early in disease progression, before substantial neuronal loss and the onset of motor, cognitive and behavioral deficits^4^.

SEMA4D plays a critical role in regulating the transition between homeostatic and reactive states of glial cells. SEMA4D binds to its plexin receptors (plexins B1 and B2) expressed on glial cells, including astrocytes, microglia, and glial progenitor cells, as well as endothelial cells, and signals through small-membrane Rho GTPases^5–7^ to regulate the actin cytoskeleton and activate NFκB, a master regulator of inflammatory cytokines^8–12^. In the central nervous system, SEMA4D is upregulated in stressed or damaged neurons and signals through plexin-B1/B2 receptors to (1) activate glial cells^13,14^; (2) disrupt normal astrocyte metabolic and synaptic functions, including glucose and glutamate transport (E.E.E. et al., in press, J Neuroinflammation, SEMA4D is upregulated in neurons of diseased brains and triggers astrocyte reactivity); (3) inhibit migration and differentiation of glial progenitor cells that can replace damaged oligodendrocytes and replenish astrocytes^5,6,15,16^; and (4) disrupt endothelial tight junctions that are required for the integrity of the blood–brain barrier (BBB)^13^. Glial cells are increasingly recognized for their important contribution to the onset and progression of neurodegenerative diseases^17^. Given the multifaceted mechanism of action of SEMA4D and the crosstalk between affected glial cells, blockade of this pathway may prevent loss of normal astrocytic functions, reprogram microglia, promote myelin repair and restore vascular changes associated with neuronal dysfunction and degeneration in HD^4^. We previously reported that an anti-SEMA4D antibody ameliorated neuropathological brain atrophy and reduced anxiety-like behavior and cognitive deficits in YAC128 HD knock-in mice^10^.

Based on the mechanism of action, activity in preclinical models of HD and other neurological diseases^10,13^ and a favorable safety profile in previous clinical studies^18–20^, the SIGNAL study was initiated to evaluate prespecified measures of safety, tolerability and potential efficacy of pepinemab as a potential therapy for HD. Efficacy endpoints were selected based on previous natural history data from the PREDICT-HD and TRACK-HD studies^21–23^, as well as results of a completed randomized pilot study in HD (cohort A, 15 EM and 21 LP participants). Magnetic resonance imaging (MRI) has been used extensively as a measure of brain atrophy in HD^24^. Fluorodeoxyglucose–positron-emission tomography (FDG–PET) detects changes in glucose transport and is a measure of overall brain metabolic activity. Decline in FDG–PET standardized uptake value ratio (SUVR) has previously been shown to correlate with cognitive and functional decline during HD disease progression^25–27^, and correlates with cognitive decline and clinical progression in other neurodegenerative diseases^28,29^.

Herein we report the results of the SIGNAL cohort B study in 265 participants known to have ≥36 cytosine-adenine-guanine repeats in one huntingtin gene and total functional capacity (TFC) 11–13, with HD diagnostic confidence level (DCL) 4 (EM) or DCL 2–3 (LP). DCL is determined by clinician assessment of motor function on the Unified Huntington’s Disease Rating Scale (UHDRS): DCL4 is defined as motor abnormalities that are unequivocal signs of HD while DCL2 and 3 are defined, respectively, as motor signs that may be (50–89% confident) or are probable (90–98% confident) signs of HD. Although the study did not meet its coprimary clinical efficacy endpoints, a favorable safety profile, significant changes in imaging measures and multiple prespecified exploratory assessments supporting cognitive benefit to patients encourage further development. The organization of data presented here is, first, to focus on prespecified endpoints stipulated in the hierarchical testing plan and, second, to report prespecified endpoints of special interest, including imaging measures of brain atrophy and metabolic activity and, given that cognitive decline is of particular concern to patients and their families, the HD–cognitive assessment battery (HD–CAB) Index. Finally, because of its importance in guiding the design of a subsequent phase 3 study, we present key post hoc subgroup analyses in support of the hypothesis that patients at a mildly advanced stage of disease, as defined below, may be more responsive to treatment with pepinemab.

## Results

### Study design and baseline demographics

The SIGNAL phase 2 trial was designed to determine whether treatment with a SEMA4D-blocking antibody, pepinemab, is safe and well-tolerated in participants with EM or LP HD, and to assess efficacy in the EM cohort, as determined by cognitive, global (CGIC), functional and neuroimaging measures. The SIGNAL cohort B phase 2 study enrolled 179 EM participants (cohort B1) with diagnosed HD (DCL 4) and with minimal to modest functional impairment (TFC 11–13), and 86 LP participants (cohort B2) with DCL 2 or 3 and cytosine-adenine-guanine (CAG) repeat/age product (CAP) score ≥200. Cohorts B1 and B2 were independently randomized 1:1 to either pepinemab (PEPI), administered intravenously (i.v.) every four weeks at a dose of 20 mg kg^–1^, or placebo (PBO) for 18 months of treatment followed by ~3 months of safety follow-up. Participants were enrolled at 30 clinical sites in the United States and Canada from 28 December 2015 to 31 December 2018. Participants, caregivers and clinician raters were blinded to treatment. Database lock and unblinding occurred in September 2020.

**Extended Data Fig. 1:**
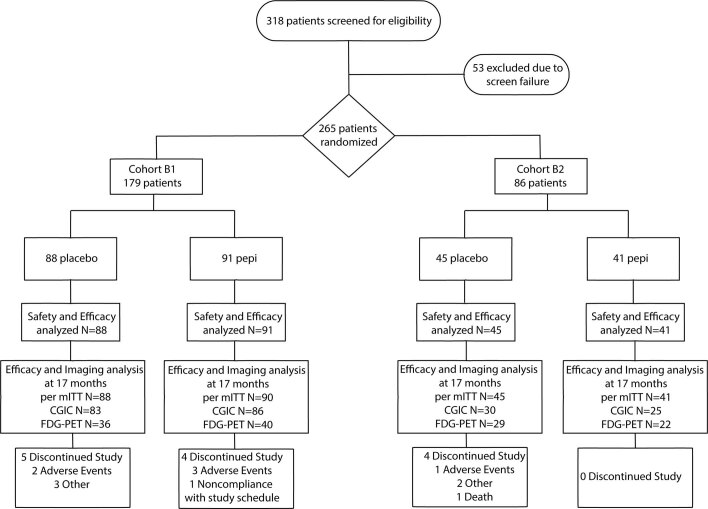
CONSORT diagram indicating participant numbers and disposition.

Study completion was high, with only 13/265 (*n* = 9 PBO, *n* = 4 PEPI) study discontinuations, as shown in the CONSORT diagram (Extended Data Fig. 1). There was limited impact of the Covid-19 pandemic, which occurred late in the study and that, as described in greater detail in Methods, was accommodated by allowing some flexibility in data collection at clinical study sites. Demographics and baseline characteristics were generally balanced between the treatment groups with respect to age, age of onset and other clinical measures obtained at screening or baseline (Extended Data Table 1 and Supplementary Fig. 1).

### Safety and tolerability

Table 1 is a summary of treatment-emergent adverse events (TEAEs). No clinically significant group differences were observed in the frequency of participants experiencing ≥1 TEAE (*P* = 0.57, 0.12 and 0.81 for B1, B2 and B overall, respectively), TEAEs of grade ≥3 (*P* = 0.7, 0.57 and 0.52) or serious adverse events (*P* = 0.24, 0.68 and 0.16). During the safety analysis period (up to 18 months of treatment), fewer subjects overall had at least one TEAE considered probably or definitely related to study drug in the placebo group compared with pepinemab (14% versus 25%, respectively). This difference was predominantly in certain mild (grades 1 and 2) TEAEs in pepinemab-treated subjects, among which headache was most frequent. One suicide death was observed in an individual with EM disease following pepinemab treatment, but was deemed unrelated to treatment.

**Table 1:** Overview of TEAEs for the safety analysis period

Category	Cohort B1, *n* = 179		Cohort B2, *n* = 86		Cohort B, overall *n* = 265	
	PBO, *n* = 87	PEPI, *n* = 92	PBO, *n* = 45	PEPI, *n* = 41	PBO, *n* = 132	PEPI, *n* = 133
Subjects with ≥1 TEAE, *n* (%)^a^	82 (94%)	84 (91%)	41 (91%)	41 (100%)	123 (93%)	125 (94%)
Probably or definitely related^b^	10 (11%)	16 (17%)	9 (20%)	17 (41%)	19 (14%)	33 (25%)
Study drug discontinuation	2 (2%)	5 (5%)	1 (2%)	0	3 (2%)	5 (4%)
Serious TEAE	8 (9%)	4 (4%)	4 (9%)	2 (5%)	12 (9%)	6 (5%)
Probably/definitely related^b^	0	0	0	0	0	0
Grade ≥3 TEAE^c^	14 (16%)	17 (18%)	6 (13%)	8 (20%)	20 (15%)	25 (19%)

Column header counts are the number of subjects in the safety population; TEAEs are those that occurred during the safety analysis period with onset on or after the date of the first exposure to study drug (PEPI or PBO).

^a^Subjects are counted at most once in each cell, regardless of the number of events they may have had; denominators are the number of subjects in the safety population, unless otherwise specified.

^b^Relatedness to study drug is defined as any of attribution of probable or definite as reported by the investigator.

^c^Grade ≥3 refers to any severe, life-threatening or fatal event.

Extended Data Table 2 shows the percentage of most common TEAEs (>10%). Most TEAEs were considered mild to moderate in severity by the investigator. The percentage of subjects with a TEAE considered severe (Common Terminology Criteria for Adverse Events (CTCAE) grade ≥3) was marginally higher for pepinemab- than placebo-treated subjects (19% versus 15%, *P* = 0.52). For each treatment group, the frequency of severe TEAEs was ≤5% within each system organ class. Results were consistent in a subset of patients evaluated for a total of 36 months of treatment and safety follow-up (Extended Data Table 2).

Immunogenicity was evaluated by measuring the presence of anti-drug antibodies (ADA). Only eight subjects (*n* = 0 PBO, *n* = 8 PEPI) were confirmed positive for ADA, and all responses were transient and of low titer (<20). No relationship between immunogenicity status and safety outcomes was observed. No subject exhibited symptoms of cytokine release syndrome during the study. Three pepinemab- and two placebo-treated subjects had six TEAEs of infusion site reaction or infusion-related reaction, but none were considered serious, led to permanent discontinuation of the study drug, nor resulted in early study withdrawal. There were no clinically meaningful changes in any hematology, serum chemistry, urinalysis, vital signs or electrocardiogram (ECG) parameters over time. No clinically notable differences in HADS anxiety or suicidal ideation (Columbia–Suicide Severity Rating Scale) were observed between pepinemab- and placebo-treated subjects.

### Efficacy

The coprimary efficacy measures for EM cohort B1 were a cognitive assessment family consisting of OTS and PTAP, two of the six components of the HD–CAB index, and the CGIC score evaluated by the treating physician. A summary of results from prespecified hierarchical testing of coprimary and secondary endpoints for cohort B1 is shown in Table 2. We had planned to analyze other UHDRS performance scales, as well as efficacy analysis of the combined B1 and B2 population (B-pooled) if the coprimary endpoints were successful. Since that was not the case, results for B-pooled are presented primarily for safety parameters in this report.

### Efficacy: cognition

The HD–CAB is designed specifically for use in clinical studies of individuals with early HD^30^ and offers sufficient range for detection of both symptomatic improvement and slowing of decline. The HD–CAB consists of six performance measures of different cognitive domains, including executive function, attention, memory, visuospatial processing, timing and emotion processing: OTS^31^, PTAP^32^, the symbol digit modalities test (SDMT)^33^, the emotion recognition test (EMO)^34,35^, the Hopkins verbal learning test–revised (HVLT-r)^36^ and the trail-making test–part B (TMT-B)^37^. Although the HD–CAB index derived from the average *z*-scores of the six components above was determined to be the most sensitive cognitive endpoint based on analysis of the pilot cohort A data, US regulators did not favor composite scores and recommended that we instead employ a smaller cognitive family evaluated without resorting to the use of *z*-scores. Results of the pilot cohort A study indicated that OTS and PTAP had the highest effect sizes (0.43 and 0.38, respectively) of the six HD–CAB components. This was consistent with the relative HD–CAB component effect sizes reported by Stout et al.^30^ Further, OTS and PTAP correlated with HD–CAB index (Pearson correlation coefficient for the change from baseline at 18 months, *r* = −0.24, *P* = 0.034 and *r* = 0.57, *P* < 0.001, respectively). We therefore selected a two-member cognitive family consisting of OTS and PTAP as a prespecified coprimary endpoint for cohort B1 evaluated using the Hochberg procedure^38^ and, as discussed below, retained the HD–CAB index as an exploratory endpoint.

**Fig. 1:**
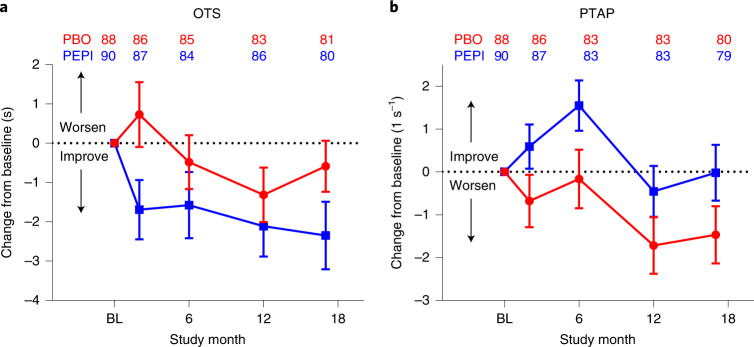
Effects of pepinemab treatment on primary cognitive assessments in EM cohort B1. **a**,**b**, Observed mean changes from baseline (BL) by treatment group over time for the mITT sample of EM cohort B1. **a**, OTS measures time to a correct response (averaged over all trials per visit). **b**, PTAP measures tapping consistency as the reciprocal of the average standard deviation of inter-tap interval durations following cessation of aural cues (over all trials per visit). **a**,**b**, Error bars show one standard error on either side of the mean, with sample sizes at each time point for each group listed above the profile lines.

In the two-member cognitive family, OTS, a test of executive function, assesses both spatial planning and working memory^39^ while PTAP, an assessment of timing and psychomotor coordination, assesses the ability to maintain a preset rhythm by consistent inter-tap intervals in the absence of continuing aural cues. EM individuals treated with pepinemab demonstrated, on average, improved performance in OTS over the 18-month treatment duration (Fig. 1a and Table 2). Improvement is reflected in lower scores in this assessment (reduced latency to correct answer in seconds), and the change from baseline at month 17 resulted in least-squares (LS) mean difference (95% confidence interval (CI)) = −1.98 (−4.00, 0.05) (one-sided *P* = 0.028). Similarly, change from baseline at month 17 in PTAP (improvement reflected in higher scores) resulted in LS mean difference (95% CI) = 1.43 (−0.37, 3.23) (one-sided *P* = 0.06; Fig. 1b and Table 2). Because neither OTS nor PTAP attained the significance threshold of one-sided *P* = 0.025, and neither member independently met the higher standard of *P* = 0.0125 required by the prespecified Hochberg analysis^38^, the results did not meet the prespecified coprimary hypothesis that pepinemab is superior to placebo in EM cohort B1 for this two-member cognitive family.

**Table 2:** Results from hierarchical testing of coprimary and secondary endpoints

Population^a^	Endpoint analysis^b^		PBO, *n*		PEPI, *n*		LS mean diff. (95% CI) PEPI versus PBO		One-sided *P* value		Favors PEPI^c^	
**Coprimary efficacy endpoints**												
Cohort B1 mITT	Two-item cognitive family	OTS (s)^d^		88		90		−1.98 (−4.00, 0.05)		0.028		(+)
		PTAP (1 s^–1^)^e^		87		89		1.43 (−0.37, 3.23)		0.060		(+)
Cohort B1 CGIC	CGIC^f^			83		84		0.06 (−0.24, 0.37)		0.35		(+)
**Secondary efficacy endpoints**												
Cohort B1 mITT	Q-motor tap speed IOI duration (ms)^g^			87		89		−1.44 (−24.26, 21.38)		0.46		(+)
	UHDRS–TFC^h^			88		88		−0.29 (−0.81, 0.23)		0.87		(−)

^a^The cohort B1 mITT population comprised 178 participants (PBO, 88; PEPI, 90) while the CGIC population comprised 169 participants (PBO, 83; PEPI, 86). The number of subjects included in an analysis may differ from the full analysis population (for example, mITT or CGIC) size if any subjects do not have both baseline (if applicable) and post-baseline observation.

^b^Analysis results are from an MMRM of change from baseline values unless otherwise specified, with estimation of the difference in means between groups at month 17.

^c^The sign next to the *P* value indicates whether the direction of the estimated difference indicates better (+) or worse (–) performance for PEPI relative to PBO.

^d^Time to a correct response (averaged over all trials per visit); lower values indicate better performance.

^e^Tapping consistency measured as the reciprocal of the average standard deviation of inter-tap interval (ITI) durations (over all trials per visit); higher scores indicate better performance.

^f^A seven-point Likert scale, ranging from very much worse (−3) to very much improved (+3); values are set to −3 following deaths adjudged by a blinded data review committee to be related to HD.

^g^IOI duration in the more-affected hand at baseline (averaged over all trials per visit); lower values indicate better performance. The more affected hand at baseline is defined for each outcome as the side with the worse score at baseline; if both hands have the same baseline score, the more affected hand will be the self-reported dominant hand; if the dominant hand is mixed, the right hand will be reported. If a subject has a baseline score for only one hand, that hand will be the more affected hand.

^h^Score ranging from 0 to 13; higher scores indicate better functioning.

### Efficacy: clinical global impression of change

The second coprimary hypothesis was that pepinemab would be superior to placebo in EM cohort B1 with respect to CGIC score at month 17 relative to baseline. CGIC is considered meaningful because it is a generic global measure^40^ consisting of a single-item assessment of the individual’s overall disease status from the clinician’s perspective based on a seven-point Likert scale. This coprimary endpoint was not met because the overall treatment effect did not reach the critical one-sided *P* value (Table 2).

### UHDRS scales and motor activity

As shown in Table 2 and further detailed in Extended Data Table 3, treatment effects on UHDRS–total functional capacity (TFC) and measures of motor activity, including UHDRS–total motor score (TMS) and multiple separate quantitative measures of motor activity (Q-motor)^41^, were not significant.

### MRI measures of brain atrophy

**Fig. 2:**
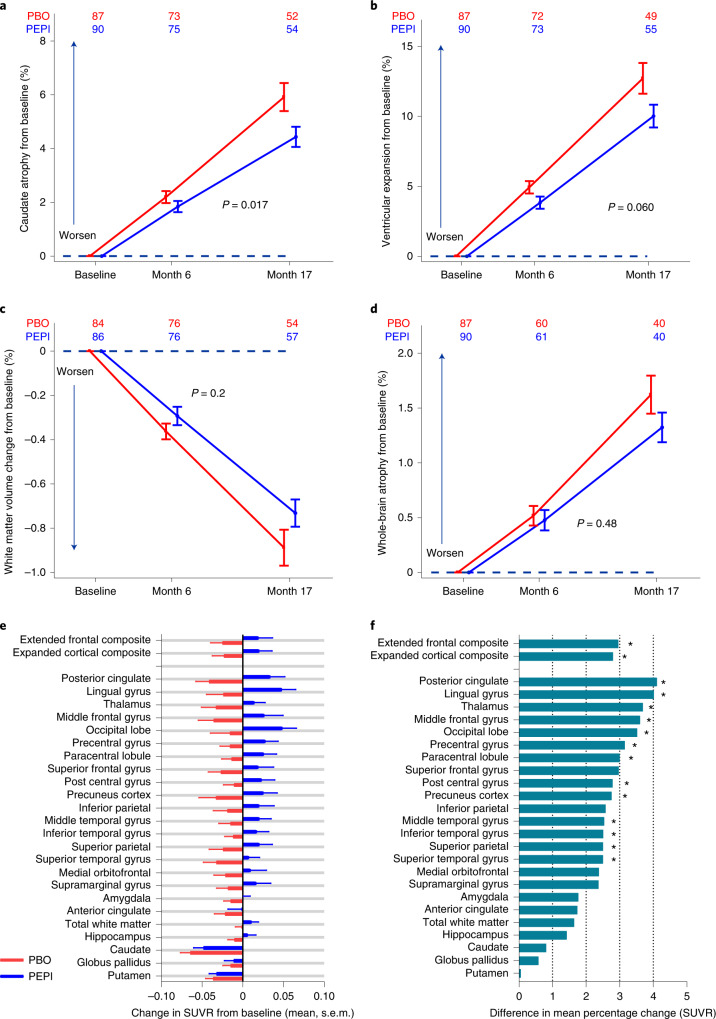
Pepinemab delays brain atrophy and restores loss of metabolic activity in EM subjects. **a**–**d**, Mean percentage changes from baseline by treatment group over time for the mITT sample of EM cohort B1 (PEPI, *n* = 90: PBO, *n* = 88) in vMRI measurement. **a**, Caudate BSI (atrophy); **b**, ventricular BSI (expansion); **c**, white matter (preservation); **d**, whole-brain BSI (atrophy). **e**, FDG–PET SUVR change from baseline to month 17 for each treatment group (mean and 1 s.d.) in each brain ROI for EM cohort B1. **f**. Treatment effect at month 17 calculated as difference between pepinemab (*n* = 40) and placebo groups (*n* = 36) as mean percentage change in SUVR. *P ≤ 0.05; exact two-sided *P* values for 15 brain regions (listed from top to bottom) are: extended frontal composite, 0.031; expanded cortical composite, 0.028; posterior cingulate, 0.008; lingual gyrus, 0.014; thalamus, 0.011; middle frontal gyrus, 0.033; occipital lobe, 0.029; precentral gyrus, 0.010, paracentral lobule, 0.014; post central gyrus, 0.028; precuneus cortex, 0.048; middle temporal gryus, 0.044; inferior temporal gyrus, 0.033; superior parietal, 0.050; superior temporal gyrus, 0.037; *P* values for all regions are shown in Extended Data Table 5. Analysis results were determined from MMRM of scheduled measurements at months 2, 6 and 17, with estimation of the difference in means between groups at month 17. *P* values are indicated (two-sided); as described in Methods, stated *P* values for all statistical tests, besides the coprimary efficacy analyses, were not corrected for multiplicity and are thus presented as nominal and not under alpha control. Error bars show one standard error on either side of the mean, with sample sizes at each time point for each group listed above the profile lines.

Volumetric MRI (vMRI) as a measure of brain atrophy was evaluated as a key secondary objective. Changes in brain vMRI (ml, %) detected by boundary shift integral (BSI)^42^, an analytic method employed in other HD studies^21–23,43^, showed a nominally significant 26% reduction in caudate atrophy for EM subjects (PEPI, *n* = 90; PBO, *n* = 88) treated with pepinemab relative to placebo, and the percentage change from baseline at month 17 resulted in LS mean difference (95% CI) = −1.54 (−2.79, −0.29) (two-sided *P* = 0.017; Fig. 2a and Extended Data Table 4). During that same period, the percentage change in ventricular volume BSI resulted in LS mean difference (95% CI) = −2.47 (−5.04, 0.10) (two-sided *P* = 0.06; Fig. 2b). Changes in the same direction were observed in EM subjects for reduction in white matter volume (Fig. 2c) and whole-brain atrophy (Fig. 2d) (Extended Data Table 4) but were not significant. LP participants (cohort B2: PEPI, *n* = 41; PBO, *n* = 45) did not demonstrate meaningful differences between drug and placebo in vMRI during the 18-month treatment duration in any of these same brain regions (Extended Data Table 4).

### Pepinemab treatment may reverse decline in FDG–PET SUVR

Change from baseline to month 17 in FDG–PET SUVR was assessed as a prespecified secondary endpoint in 23 brain regions of interest (ROIs), plus total white matter and two composite regions of extended frontal and additional cortical brain regions (ROIs and composite regions listed in Methods). Subsets of EM subjects (*n* = 40 PEPI, *n* = 36 PBO) and LP subjects (*n* = 22 PEPI, *n* = 29 PBO) were analyzed for FDG–PET imaging, dependent on the ability to perform imaging at each site and subsequent imaging quality control. The longitudinal change in SUVR was calculated for each EM subject in each ROI, and group mean changes are plotted in Fig. 2e. As expected from previous natural history studies, SUVR declined across all ROIs in the placebo group and this is also reflected in the composite regions. In striking contrast, an increase in SUVR was observed in almost all ROIs following treatment with pepinemab, with the exception of regions of the basal ganglia including striatum (caudate, putamen) and globus pallidus. The difference in mean percentage change in SUVR between pepinemab and placebo is plotted by rank order in Fig. 2f and listed alphabetically in Extended Data Table 5a. The estimate of the difference between pepinemab and placebo in percentage change from baseline in FDG–PET SUVR was quantitatively beneficial for pepinemab over placebo for all 26 regions in EM cohort B1, and significant for 15 regions (*P* ≤ 0.05). These data suggest that, after 18 months of treatment in EM subjects, pepinemab prevents and/or reverses the decline in metabolic activity in the majority of brain regions evaluated, particularly in the frontal and cortical regions.

### Exploratory cognitive measures

**Fig. 3:**
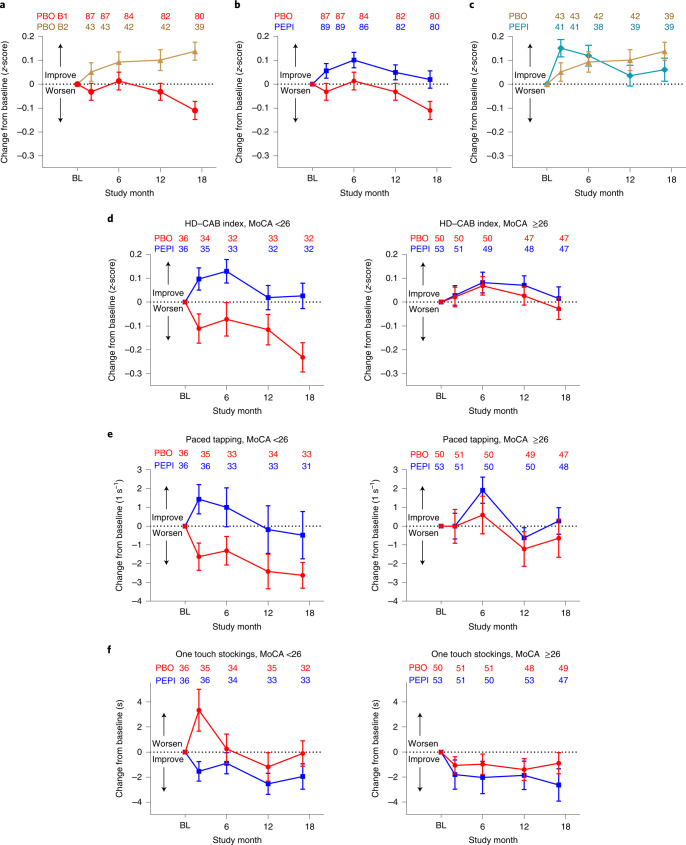
Exploratory cognitive measures and post hoc subgroup analysis of baseline MoCA as a biomarker for treatment response in early HD. **a**–**c**, Observed mean changes from baseline by treatment group over time for the placebo groups alone of cohort B1 (red circles) and B2 (brown triangles) (**a**), for both placebo and pepinemab treatment groups of EM cohort B1 (**b**) and LP cohort B2 (**c**). Cognitive assessments stratified by baseline MoCA scores of <26 and 26–30 for assessments of HD–CAB index (**d**), PTAP (**e**) and OTS (**f**). Error bars in each panel show one standard error on either side of the mean, with sample sizes at each time point for each group listed above the profile lines. PEPI (blue squares) and PBO (red circles) in EM cohort B1, and PEPI (teal diamonds) and PBO (brown triangles) in LP cohort B2.

Given the importance attached by patients and their families to cognitive decline during disease progression^1,2^, we investigated whether the HD–CAB index, a prespecified exploratory endpoint previously employed in observational studies, could provide additional information regarding cognitive decline. Each of the six HD–CAB components individually showed change in the direction of benefit favoring pepinemab over placebo for EM cohort B1 (Extended Data Table 6) and, under these conditions, the mean difference in HD–CAB index between drug and placebo, which is an average of the individual *z*-scores, can be substantially more significant than that of the individual component scores due to reduced standard error. The HD–CAB index change from baseline at month 17 (95% CI) resulted in LS mean difference (95% CI) = 0.13 (0.03, 0.23) (one-sided nominal *P* = 0.007; Fig. 3 and Extended Data Table 6). In a post hoc sensitivity analysis of EM cohort B1, the same analyses were repeated on HD–CAB index modified by omission of OTS, resulting in LS mean difference (95% CI) = 0.12 (0.00, 0.23) (nominal one-sided *P* = 0.023), omission of PTAP (LS mean difference (95% CI) = 0.09 (0.01, 0.18) nominal (one-sided *P* = 0.019)) or omission of both OTS and PTAP (LS mean difference (95% CI) = 0.10 (−0.03, 0.22) (nominal one-sided *P* = 0.064)). These data highlight that the HD–CAB index captures the trend of change in HD–CAB assessments beyond the significance of just the one or two leading individual assessments.

An important consideration for evaluation of cognitive performance is the role of ‘learning effects’ during early evaluation sessions. It has previously been reported for Alzheimer’s disease that reduced learning effects are associated with increased levels of biomarker burden and greater cognitive decline^44,45^. A comparison of change in HD–CAB index for EM cohort B1 and LP cohort B2 subjects treated with placebo only (Fig. 3a) suggests that there is a greater learning effect for LP than for EM subjects. Importantly, pepinemab treatment preserves this learning ability in EM subjects (Fig. 3b,c). Because the ability to learn is itself an important cognitive function, this suggests that improvement in the HD–CAB index of EM subjects during the first months of treatment with pepinemab may be due to preservation of learning effects that are otherwise lost in EM patients. Treatment effects do, however, appear to continue through 18 months of exposure as detected by longitudinal changes in MRI (Fig. 2a–d), and are also suggested by increasing separation in HD–CAB index between drug and placebo for months 12–17 (Fig. 3b,d).

Finally, in regard to other changes related to cognition, the apathy severity subscore of the problem behaviors assessment (PBA-s) has previously been reported to correlate with cognitive deficits during early HD progression^46^. In a post hoc analysis of the PBA-s apathy severity subscore for EM cohort B1, the proportion of subjects with increased apathy severity at month 17 was nominally significantly lower in the pepinemab group compared with placebo (23% PEPI versus 40% PBO), with an odds ratio (95% CI): 0.46 (0.22, 0.94), one-sided *P* = 0.017. Subscores for the three other most prevalent problematic behaviors in HD—depressed mood, anxiety and irritability—did not show significant change from baseline at month 17 (Extended Data Table 7). Treatment-related changes in PBA-s subscores including apathy were not evident in LP cohort B2.

### Imaging and cognitive effects in LP cohort B2

The SIGNAL study tested a clinical intervention in both a prodromal and a manifest HD population. Cohort B2 enrolled LP subjects to explore which, if any, outcomes might indicate efficacy signals in this population. As reported in Extended Data Table 4, there was no evidence of a significant treatment effect on caudate atrophy (*n* = 78) or ventricular volume (*n* = 75) in LP subjects. The magnitude of changes in FDG–PET SUVR for LP cohort B2 (Extended Data Table 5b) was much reduced compared with EM cohort B1 (Extended Data Table 5a). The average percentage change between baseline and month 17 across all 26 indicated brain ROIs for the LP B2 placebo group was −0.21%, and for B1 was −1.79%. Further, no significant treatment effect was observed in any brain ROI for B2 (Extended Data Table 5b) whereas *P* ≤ 0.05 for B1 in 15 of 26 ROIs (Fig. 2f and Extended Data Table 5a). Similarly, for LP cohort B2, treatment-related changes in individual-component HD–CAB cognitive assessments were not consistent in either magnitude or direction, and the difference in HD–CAB index between drug and placebo was not significant for this prodromal population, with LS mean difference (95% CI) = −0.11 (−0.27, 0.06) (nominal one-sided *P* = 0.90; Extended Data Table 6 and Fig. 3c). We discuss below how this may be consistent with our understanding of a principal mechanism of action of SEMA4D blockade on glial cell reactivity.

### Post hoc analysis: contribution of baseline disease state

It became evident in the analysis of treatment outcomes that some effects of pepinemab treatment were detectable in EM but not in LP subjects. We therefore tested the hypothesis that the ability to detect change over the 18-month study duration might be improved in participants already showing deficits at baseline: an important consideration for clinical trial design. We first investigated whether cognitive change in HD–CAB is differentially detectable in subjects with early signs of cognitive decline. The Montreal Cognitive Assessment (MoCA) is a validated rapid-screening instrument for assessment of cognitive dysfunction, and was administered to determine subject eligibility based on an exclusion criterion of MoCA score ≤22. The maximum possible MoCA score is 30 points; a score of ≥26 is considered normal and a score <26 indicates some degree of cognitive deficit. Using MoCA as an independent measure of cognition, we performed post hoc analyses to stratify outcomes of HD–CAB by baseline score of MoCA <26 or ≥26. Results for the HD–CAB index, PTAP and OTS are shown in Fig. 3d–f, and for the other HD–CAB components in Supplementary Fig. 2. The LS mean difference between treatment arms for HD–CAB index in the MoCA ≥26 subgroup was not significant. In contrast, in participants with MoCA <26 at baseline there was a steady decline in HD–CAB scores in the placebo group during 18 months of treatment, while the HD–CAB index in the pepinemab group did not fall below baseline at any time point (Fig. 3d). The change from baseline at month 17 in the MoCA <26 subgroup resulted in a LS mean difference (s.e.) of 0.24 (0.08) (nominal one-sided *P* = 0.0025). Similarly, for PTAP (Fig. 3e), the LS mean difference (s.e.) in the MoCA <26 subgroup was 1.89 (1.10) (nominal one-sided *P* = 0.044), compared with no significant change in the MoCA ≥26 subgroup with LS mean difference (s.e.) of 1.09 (1.32) (one-sided *P* = 0.21). Stratification by baseline MoCA score, however, does not have a similar impact on mean difference and *P* value for OTS (Fig. 3f). It appears that decline in OTS begins earlier in disease progression than for other HD–CAB domains: LS mean difference (s.e.) = −1.87 (1.44) (one-sided *P* = 0.099) for MoCA <26 and, for MoCA ≥26 subgroups = −1.73 (1.34) (one-sided *P* = 0.101). Indeed, because of the smaller group sizes (*n* = 32 and 47), this resulted in less significant one-sided nominal *P* values (*P* = 0.099 and 0.101) relative to the total cohort B1 population (*n* = 79, *P* = 0.028). These results suggest that HD–CAB is an effective instrument for capturing changes across different cognitive domains that may decline at different rates during disease progression.

**Fig. 4:**
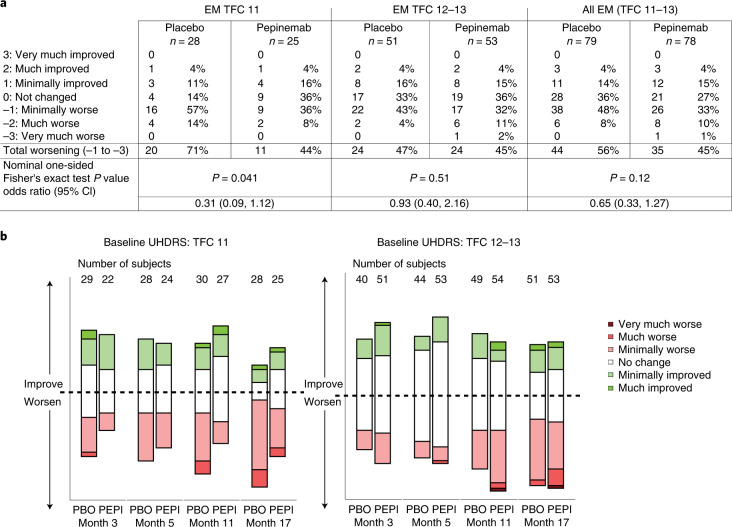
Effects of pepinemab treatment on CGIC in EM cohort B1. **a**, Observed categorical CGIC values at visit 17 for the entire EM cohort B1 CGIC analysis population, and in two subgroups stratified by baseline TFC value (that is, 11 and 12–13). **b**, Observed categorical CGIC in the subgroups stratified by TFC value over the study duration. The CGIC is a seven-point Likert scale, ranging from very much worse (−3) to very much improved (+3). Values were set to −3 following a patient death adjudged by a blinded data review committee to be related to Huntington’s disease. *P* values determined by one-sided Fisher’s exact test; odds ratio (95% CI) are also shown.

We next considered the possibility that a treating physician’s ability to detect change, as reflected in CGIC, might be greater in subjects with somewhat more advanced disease. UHDRS–TFC is a 13-point, standardized disease-staging scale that rates a person’s functional capacity and level of independence in five domains: occupation, ability to manage finances, ability to perform domestic chores, ability to perform personal activities of daily living and level of care^47^. Higher scores indicate higher functioning capacity, with a maximum score of 13. Subjects with TFC 11 would, therefore, be expected to have more advanced presentation of symptoms than those enrolling at the highest end of the scale, TFC 12–13. Using TFC as an independent functional measure to stratify CGIC scores, among the subgroup of patients with baseline TFC 11, significantly fewer subjects had a worsened disease CGIC status in the pepinemab group: specifically, 44% PEPI versus 71% PBO, with an odds ratio (95% CI) of 0.31 (0.09, 1.12) (nominal one-sided *P* = 0.041), as compared with 45% PEPI versus 47% PBO for the TFC 12–13 subpopulation of EM cohort B1 (Fig. 4). Although the TFC 11 subgroup sample size is small, this is consistent with the hypothesis that potentially greater treatment benefit can be detected in subjects with somewhat more advanced disease and could be an important consideration in the design of subsequent studies.

### Pharmacokinetics/pharmacodynamics

**Extended Data Fig. 2:**
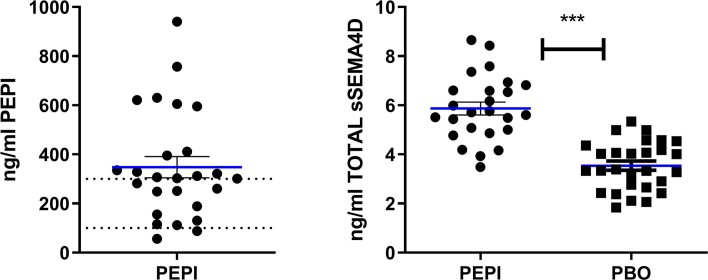
Pepinemab is detected in CSF at expected level for target engagement. **a**. Drug concentration in CSF of subjects treated with pepinemab. Mean+SEM is shown; dotted lines indicate target concentration (~100-300 ng/ml). **b**. Concentration of soluble SEMA4D (sSEMA4D) in CSF, mean+SEM are shown for each treatment group (n = 26 PEPI, n = 28 PBO). *** indicates statistical significance, p = 0.000000001855. As seen in previous trials^18,19^, levels of total soluble SEMA4D (including complex of drug bound to target) increased 1.7-fold upon dosing due to the increased half-life of the pepinemab/SEMA4D complex in subjects treated with pepinemab compared to those treated with placebo (average of 5.9 vs 3.5 ng/ml respectively, p < 0.001), demonstrating evidence of target engagement in CSF. The mean (SD) observed maximum serum concentration (Cmax) after all infusions post visit 12 in Cohort B overall was 218 (115) µg/mL and the AUC_tau_ over the dosing interval was 68,900 (14,447) µg*hr/mL. Based on the empirical Bayesian estimates of the pharmacokinetic parameters, the terminal elimination half-lives for Cohorts B1 and B2 were calculated to be approximately 25 and 23 days, respectively. In general, the clearance of pepinemab was low and the volume of distribution small, which is common to other therapeutic monoclonal antibodies and similar to observations in previously completed studies with pepinemab^18,19^.

The 20 mg kg^–1^ dose of pepinemab administered i.v. every four weeks was selected to achieve a projected dose in cerebrospinal fluid (CSF) of 100–300 ng ml^–1^, estimated as the effective range from earlier dose-titration studies. Pepinemab drug levels and total soluble SEMA4D were measured in CSF in a subset of 54 subjects (*n* = 28 placebo and *n* = 26 pepinemab) predominantly during months 13–18 (mean, 15.6; median, 15). Pepinemab was detected in CSF at a mean level (95% CI) of 348 ng ml^–1^ (220.7, 437.2) and the average CSF/serum concentration ratio of pepinemab was 0.38 (0.39)% (Extended Data Fig. 2a). No drug was detected in subjects treated with placebo, as expected. Evidence of target engagement in CSF based on increased concentration of soluble SEMA4D is reported in Extended Data Fig. 2b.

## Discussion

Predefined primary efficacy endpoints did not achieve statistical significance in the SIGNAL study. Nevertheless, a significant pepinemab treatment-related reduction in caudate brain atrophy and increase in FDG–PET SUVR in most brain ROIs, along with multiple prespecified exploratory assessments that suggest a cognitive benefit of treatment, encourage continued development of pepinemab as a potential therapy for EM HD.

In two broad surveys, patients with HD have identified cognitive decline as a major concern. Even subtle cognitive changes can affect functional abilities such as work performance, driving and financial management^21,48,49^. While predefined primary endpoints of cognition, OTS and PTAP, failed to achieve statistical significance (one-sided *P* = 0.028 and 0.06, respectively) other indications of cognitive benefit of pepinemab treatment include a nominally significant effect on the HD–CAB index (one-sided *P* = 0.007), a prespecified exploratory endpoint, as well as post hoc analysis demonstrating a nominally significant treatment effect on apathy severity (*P* = 0.017), a component of the prespecified exploratory PBA-s that has been reported to correlate with cognitive decline. It was also of particular interest that learning effects appear to be compromised early in HD progression, as previously reported for early Alzheimer’s disease^44,45^, and that pepinemab treatment appears to improve or rescue this cognitive feature. It would be desirable to add a direct assessment of ability to learn in future studies.

The SIGNAL clinical study has established the safety and tolerability of pepinemab immunotherapy in individuals with HD. No concerning clinically relevant safety issues were identified, concordant with other completed clinical studies of pepinemab in individuals with multiple sclerosis and advanced solid tumors^18–20^. One suicide death was observed in an individual with EM disease following treatment with pepinemab, but was deemed unrelated to treatment. Natural history studies report that the prevalence of suicidal ideation is a common symptom of HD and occurs in about 24% of individuals during the early stage of the disease^50^. Nevertheless, suicidal ideation and suicide attempts should be carefully monitored in future studies of pepinemab in HD.

A major goal of phase 2 studies is to identify the patient population likely to benefit from treatment. Post hoc subgroup analysis suggested a potential treatment benefit to patients who were somewhat more advanced in disease progression, as indicated by improved CGIC score in patients with a baseline TFC score of 11 relative to less advanced TFC 12–13, and also supported by increased relative response to treatment for OTS, PTAP and the HD–CAB index in patients with MoCA <26 relative to MoCA ≥26 at baseline. This hypothesis, based on post hoc analysis of relatively reduced group sizes, will require empirical testing in a future phase 3 study of pepinemab that should include a broader TFC range—for example, enrollment of patients with baseline TFC 8–12. It may naturally occur that this population will also be substantially enriched for MoCA <26, but further stratification could also be considered if that were not the case.

There are considerations related to the mechanism of action of pepinemab that may explain a delay in the ability to detect treatment-related changes very early in disease. As reported elsewhere^13^ (and in E.E.E. et al., in press Ibid), SEMA4D plays an important role in the promotion of astroglial and microglial reactivity. This entails complex interactions between astrocytes and microglia and among different subsets of each. Counterbalancing activity of different inflammatory cells may, in normal circumstances, maintain a homeostatic balance. However, under the influence of a disease-related disturbance, this process may fail to arrive at a healthy equilibrium. In a slowly progressive disease like HD it could, therefore, be a matter of time before the homeostatic process is exhausted and the benefits of treatment become evident. As discussed below, it is, of course, also possible that there are other early-stage pathogenic events unrelated to glial reactivity that are not impacted by SEMA4D blockade.

Consistent with the natural history of HD, brain atrophy progressed and metabolic activity decreased in most brain ROIs in the placebo group for EM HD. This was in contrast to a pepinemab-associated decrease in caudate brain atrophy (95% CI) of −1.54% (−2.79, −0.29), *P* = 0.017 and a trend of reduced ventricular expansion (95% CI) of −2.47% (−5.04, 0.10), *P* = 0.060. Importantly, decline in metabolic activity detected by FDG–PET is a regular feature of other slowly progressive neurodegenerative diseases, and multiple studies in Alzheimer’s disease have demonstrated that decline in FDG–PET SUVR correlates with cognitive decline and clinical progression^28,51^. The observation that treatment with pepinemab results in a significant increase in FDG–PET SUVR in most brain ROIs is a notable reversal of the previously reported decline in FDG–PET signal during HD progression^25–27^. It was unexpected that regions of the basal ganglia, including caudate and putamen, did not manifest the same degree of treatment-related increase in FDG–PET SUVR observed in most other brain ROIs of EM HD subjects. Striatal regions are known to degenerate at a rate three- to fourfold greater than other brain regions even before the time of motor diagnosis^22,52^. Loss of neurons is certain to impact energy metabolism and may be triggered by mechanisms that directly affect medium spiny neurons in striatum and are independent of SEMA4D activity and, therefore, relatively unaffected by pepinemab treatment. In keeping with the absence of a treatment effect on glucose utilization in striatum, we also did not observe an effect on early motor dysfunction—for example, chorea—in either EM or LP populations as detected by either UHDRS–TMS or Q-motor assessments. It is possible that later stages of motor progression could be affected by treatment but are less prominent in the TFC ≥11 population.

As might be expected, given their important role in glucose transport, recent reports demonstrate that astrocytes contribute substantially to the FDG–PET signal in the brain^53^. Normal astrocytes extend numerous cytoplasmic projections with endfeet that express glucose transporter and fully cover brain capillaries. These projections are reduced and glucose transport is downregulated in reactive astrocytes^54,55^. SEMA4D is known to trigger collapse of the cell cytoskeleton and to downregulate glucose transporters on astrocytes, while SEMA4D blockade restores glucose uptake (E.E.E. et al. in press, Ibid) and may, therefore, counteract decline in FDG–PET signal associated with astrocyte reactivity. There may be other potential effects of SEMA4D blockade that could impact FDG–PET. We have previously reported that SEMA4D blockade protects the integrity of the BBB^13^. Compromise to the BBB could have a profound impact on transport and synaptic activity that may impact energy metabolism. We considered the possibility that changes in FDG–PET SUVR might reflect an undesirable increase in inflammation, but evidence of our own and from others^13,56^ suggests that the effects of SEMA4D antibody blockade are anti-inflammatory. For example, we previously reported that SEMA4D antibody prevents inflammatory activation of murine Iba-1^+^ microglia^13^, and others have recently demonstrated that the SEMA4D–plexin-B1/B2 signaling pathway governs crosstalk between astrocytes and microglia that triggers glial activation^56^. SEMA4D blockade should, therefore, be expected to reduce inflammation. These novel findings suggest that FDG–PET may hold promise as an early biomarker of potentially effective treatments in HD.

In parallel with metabolic effects in multiple cortical regions, there appeared to be a clear effect of pepinemab treatment in EM disease on OTS (one-sided *P* = 0.028), a measure of executive function that is a key cognitive domain related to learning, as well as on direct learning effects evident during sequential HD–CAB assessments. In contrast to Alzheimer’s disease, for which the clinical dementia rating scale and Alzheimer’s disease assessment scale-cognitive subscale are well established as meaningful composite and cognitive endpoints, there is no established measure of cognition in HD that is currently accepted as intrinsically meaningful by both US and European Union regulators. We suggest that direct measures of learning could constitute an intrinsically meaningful cognitive endpoint in manifest HD.

Important limitations of the SIGNAL study design include exclusion of patients with TFC <11. This was highlighted by post hoc subgroup analysis which suggested that pepinemab treatment effects appear to be more evident in patients with early cognitive or functional deficits. While MMRM analyses provided mathematical power for independent imaging and cognitive outcomes, power for correlation analysis with imaging endpoints was limited due to further reduced group sizes associated with scheduling issues, quality control and a subset of sites unable to perform FDG–PET. Despite these limitations, key observations suggesting a trend of benefit in a two-item cognitive family, supported by significant change in prespecified exploratory analysis of the HD–CAB index, improved apathy score, a reduction in caudate atrophy and increase in metabolic activity as reflected by FDG–PET SUVR, collectively suggest clinically relevant changes in patients with early manifest Huntington’s disease. Further studies are warranted to confirm and extend these observations, with adjustments to enroll patients with early signs of cognitive or functional deficits.

## Methods

### SIGNAL study design and participants

SIGNAL cohort B is a phase 2, multicenter, randomized (1:1), double-blind, placebo-controlled, parallel-group study of pepinemab in subjects with EM (cohort B1) and LP (cohort B2) HD (https://clinicaltrials.gov/ct2/show/NCT02481674) (no. VX15-2503-N-131B). A total of 265 subjects (179 EM and 86 LP) were enrolled at 30 outpatient clinical sites in the United States and Canada. Only a subset of sites (*n* = 25) were able to perform FDG–PET imaging. All subjects received at least 17 planned monthly infusions.

Eligible participants were aged 21 years and older, had genetically confirmed presence of ≥36 CAG repeats in one huntingtin gene and no features of juvenile HD (Westphal variant). EM participants were defined by UHDRS–TFC ≥11; they were determined by the site investigator to have a clinical diagnosis of HD as defined by a DCL of 4. LP was defined as DCL of 2 or 3 and a CAP score of >200. CAP score, a measure of HD mutation burden, was calculated as the product of age and (CAG-33.66)^57^. Stable dosages of concomitant medications (including tetrabenazine and deutetrabenazine) were permitted if initiated at least 1 month before baseline (visit 0), as were newly prescribed anxiolytics for use as premedication before imaging at screening, which were permitted on a case-by-case basis. Exclusion criteria included participation in an investigational drug or device study within 30 days of baseline (or 180 days if the previous investigational drug was a monoclonal antibody therapeutic), suicide risk (as determined by the Columbia–Suicide Severity Rating Scale), MoCA score ≤22, ECG abnormalities at screening, pregnancy and conditions that would exclude MRI participation.

#### Randomization and blinding

Subjects who satisfied all eligibility criteria were participants and were randomized to one of two treatment arms through an interactive web response system. The subjects, site investigators, site personnel and study statisticians—as well as representatives of these organizations and staff at Vaccinex—were blinded as to treatment assignments until database lock. The investigational agent and placebo were in vials of identical appearance. During the course of the study, the Safety Monitoring Committee maintained access to treatment code information. Omnicomm Inc. eClinical Solutions v.5.2 was utilized for data collection on SIGNAL.

#### Investigational agent and treatment

Pepinemab (VX15/2503) is a humanized IgG4 monoclonal antibody^58^ with a hinge mutation that prevents in vivo Fab arm recombination^59^. Bulk pepinemab (Catalent Pharma Solutions) was produced using a proprietary CHO cell line. The bulk antibody was purified using standard techniques and formulated at approximately 20 mg ml^–1^ in preservative-free 20 mM sodium acetate pH 5.4, containing 130 mM sodium chloride and 0.02% polysorbate 80. Pepinemab and matching placebo were supplied by the sponsor as single-use vials. Subjects enrolled in cohort B were randomized to receive monthly i.v. 60-min infusions of either placebo or 20 mg kg^–1^ pepinemab. Selection of the 20 mg kg^–1^ i.v. dose of pepinemab for the SIGNAL cohort A study was based on findings from nonclinical studies^60^, as well as on single- and repeat-dose Phase 1 and 2 studies of pepinemab up to 20 mg kg^–1^ in subjects with multiple sclerosis^19^ and in subjects with advanced solid tumors^18^, respectively. Placebo or drug was administered for up to 18 months of treatment. Subjects were treated at baseline through to visit 17. Efficacy endpoints were assessed on visit 17 after 18 months of study drug exposure, and a month 18 visit or phone call at the end of the safety analysis period, which spans a total of 18 months. Subjects were then followed for an additional ~3 months (minimum of 2 months and up to 6 months after last infusion) for safety and laboratory assessments. Additionally, under protocol amendments 1–3, the first 53 subjects enrolled in cohorts B1 or B2 were offered the option to receive 18 additional months of treatment for a total of up to 36 months safety evaluation. From protocol amendments 4−6, the treatment period was 18 months. Of the 53 subjects who were offered entry into the extended treatment period, 42 agreed.

#### Outcome measures

Primary outcome measures were defined as: (1) to evaluate the safety and tolerability of monthly i.v. administration of pepinemab relative to placebo over 18 months of double-blind treatment in the entire study population (cohorts B1 and B2); and (2) to evaluate the efficacy of monthly i.v. administration of pepinemab relative to placebo over 18 months of double-blind treatment in EM subjects (cohort B1), including change from baseline to month 17 in the two-item cognitive family (PTAP and OTS components of the HD–CAB) and CGIC at month 17 relative to baseline. Secondary and exploratory outcome measures reported here include UHDRS–TFC and TMS, Q-motor, pharmacokinetics (PK), pharmacodynamics (PD) and immunogenicity; additional cognitive assessments, in particular the HD–CAB index; and imaging assessments, including change from baseline to visit 17 in FDG–PET SUVR and in brain volume as measured by volumetric MRI.

The ITT population consisted of all randomized subjects categorized by their randomized treatment assignment. The mITT population consisted of all randomized subjects who had received at least one complete infusion of study drug and had at least one post-infusion efficacy evaluation for one of the coprimary efficacy outcomes—that is, cognition or CGIC. The safety population consisted of subjects who had received at least one dose of study drug (including one patient who received a partial infusion); efficacy analyses was performed on the mITT population. A subset of subjects in the mITT population were assessed for CGIC, due to addition of this outcome measure in amendment 5, and a different subset were assessed by FDG–PET due to the limited number of participating sites and consenting individuals, as described in the CONSORT diagram (Extended Data Fig. 1).

#### Description of outcomes

Tolerability, defined as the ability to complete the study on the assigned study arm, accounted for subjects’ study disposition (for example, reason for study discontinuation of ‘Did not tolerate study drug’), treatment disposition and duration of exposure. Adverse events were monitored monthly for each subject during the study period, defined as from signing informed consent through to final study contact. A TEAE was defined as an adverse event with onset on or after the date of first dose of study drug. AEs were coded using the Medical Dictionary for Regulatory Activities v.16.1. The investigator assessed the causality of each AE to the study drug and the severity of each AE using his/her clinical expertise, and designated a grade to each AE according to the current CTCAE. The Columbia–Suicide Severity Rating Scale and Hospital Anxiety and Depression Scale were both assessed. The HD–CAB was measured at six visits during the primary analysis period: screening, baseline and months 2, 6, 12 and 17. The HD–CAB comprises six component tests^30^, among which are PTAP and OTS, and is described in the table below. PTAP measures the consistency of tapping rate, measured as the reciprocal of the standard deviation of ITI duration. OTS measures the mean time (in seconds) to a correct response. CGIC^40^ is a single-item questionnaire that asks the investigator to assess a subject’s HD symptoms compared with those immediately before starting the study drug, using a seven-point Likert scale ranging from very much worse (−3) to very much improved (+3), to assess overall response to the study drug relative to baseline. CGIC was evaluated at four visits during the primary analysis period: months 3, 5, 11 and 17. The TFC scale and TMS are components of the UHDRS, including UHDRS–TFC and UHDRS–TMS, and have been used in observational studies and randomized controlled trials in both premanifest and manifest HD populations^47,61,62^. The UHDRS–TFC score is the sum of five items ranging from 0 to 13, with a higher score representing better functioning. Q-motor and TMS allow objective monitoring of motor effects. The UHDRS–TMS score is the sum of scores of 31 items in four domains, the response to each being rated on a four-point scale for a maximum score of 124, with higher scores indicating more severe motor impairment^47^. The Q-motor battery is composed of precalibrated and temperature-controlled force transducers and three-dimensional position sensors that are used to assess (1) grasping forces, (2) involuntary choreatic movements, (3) regularity of index finger tapping and (4) regularity of alternating pronation/supination hand movements. PBA-s is a semistructured clinical interview that contains 11 items, each measuring a different behavioral problem rated for both severity and frequency on a five-point scale (0–4)^63^. HD–CAB component tests and variables of interest were as follows.

**Table:** 

Test name	Cognitive function assessed	Variable of interest	Value range for variable of interest
HVLT-r	Verbal memory	Total correct recall trials over three learning and one delayed recall trials; higher score indicates better performance	0–48
SDMT	Visuospatial attention, processing speed, working memory	Number of correctly coded terms; higher score indicates better performance	0–110
TMT-B*	Flexibility, visuospatial attention, psychomotor speed	Time (seconds) to complete the task; higher value indicates poorer performance	0–240
EMO	Emotion recognition	Number of negative emotions correctly identified; higher score indicates better performance	0–24
**PTAP**	**Timing, psychomotor coordination**	**Reciprocal of the standard deviation of the ITI duration that occurred following cessation of pacing tones over all trials taken; higher value indicates better performance**	**Not defined**
OTS*	**Planning, working memory**	**Mean time to reach a correct response (seconds), averaged across ten trials; higher values indicate poorer performance**	**Not defined**

Bold font indicates membership in the HD–CAB two-item cognitive family for coprimary endpoint in EM cohort B1. For tests with an asterisk (*), a lower value of the variable of interest indicates better performance; for all others, a higher value indicates better performance.

### COVID considerations

As a result of the COVID-19 pandemic declared by the World Health Organization in March 2020, and in accordance with the FDA Guidance ‘Conduct of Clinical Trials of Medical Products during COVID-19 Pandemic’, released in March 2020 and updated in June 2020, changes were made to allow flexibility in data collection at clinical study sites while maintaining participant safety and clinical trial integrity. Visit windows and visit schedule were updated to allow less time between visits and to permit key data collection planned for the final visit to be collected at an earlier or later visit within an allowed time frame (plus or minus one visit) if a site was open; if a site had closed, the site could collect these data when local guidance permitted. Subjects able to attend visit 16 or 17 received an infusion at the investigator’s discretion. The date 11 March 2020 was selected as the date on or after which the COVID-19 pandemic was likely to have influenced data collection for this study.

### MRI imaging

A subset of 178 EM subjects (90 pepinemab, 88 placebo) and 86 LP subjects (41 pepinemab, 45 placebo) participated in MRI imaging. MRI neuroimaging was scheduled at screening and months 6 and 17. IXICO, Ltd (www.ixico.com) reported volumes at screening and changes from baseline at postscreening visits according to the BSI analysis method^42^. White matter volume change was calculated with a Jacobian method. Other region segmentations were calculated with the ‘Learning Embeddings for Atlas Propagation’ (LEAP) method, both for volume measures and for use in the FDG–PET SUVR pipeline. All sites and their respective 3T MRI scanners were qualified before the start of the study. The protocol included the following scan sequences: (1), localizer; (2), T1w; (3), field map DTI; (4), DWI; (5), T2_SPACE; (6), T2_FLAIR; (7) field map functional MRI; (8), resting state functional MRI; and (9) PD, but analysis involved only analysis of volumetric changes (T1w). IXICO reports the pseudo-total intracranial volume (pTIV) factor, which is a measure of how a subject’s head size compares with the standard template. Screening results were normalized to the pTIV to account for differences in head size. The normalized screening result for each brain region was calculated as screening volume/pTIV factor. At each post-baseline time point, percentage change from baseline was calculated as (reported change value/reported screening value) × 100. MRI preprocessing software included MRIcron DICOM to NIfTI conversion (v.6/2013), DCMTK extraction of orientation information (v.3.5.4), image registration toolkit (IRTK, v.1.95) for multiatlas brain extraction and registrations, N4BiasFieldCorrection (v.1.9 from the ANTs package), MIDAS for semiautomated delineation (v.5.11.1), KN-BSI for boundary shift integral calculation of volume change (v.1.2), NiftyReg/NiftiSeg (v.0.9.4) and NifTK for BSI computation (v.12.11).

### FDG–PET

A subset of 76 EM subjects (40 pepinemab, 36 placebo) and 51 LP subjects (22 pepinemab, 29 placebo) participated in FDG–PET imaging. ^[18]^-Fluoro-2-deoxy-d-glucose was employed as ligand in FDG–PET. FDG–PET imaging was performed at screening and again post baseline, approximately at months 2, 6 and 17. Primary FDG–PET scans were analyzed without knowledge of treatment assignments by IXICO. For imaging data used for this investigation in regard to PET images specifically preprocessed with the IXICO PET Regional SUVR pipeline, PET dynamic average was registered to native 3D–T1w space and WB–LEAP segmentation^64–66^ of the same subject to allow for regional uptake sampling. FDG–PET images underwent image quality control. All FDG–PET images that passed image quality control were entered into the quantitative SUVR workflow for computation of SUVR for a number of brain regions, using the pons as the reference region. This involved computation of regional PET tissue activity values, correcting for radioactive decay and patient weight and standardizing according to a tracer-specific reference region, to allow comparison between patients. Brain regions for which SUVR was calculated were found using LEAP with the subject 3D–T1w MR image, which is registered to the FDG–PET image. SUVR quantification was performed on standard FDG–PET images acquired within 30-min intervals, 40 min after injection. FDG–PET data for the left and right hemispheres were averaged because within-subject changes in both hemispheres were strongly correlated (*r* = 0.98, *P* < 0.001). Prespecified brain ROIs (*n* = 26) were analyzed; additionally, composite SUVRs were computed using the mean SUVR in the masked region consisting of the following regions that comprise (1) the extended frontal composite: anterior orbital gyrus, central operculum, frontal operculum, postcentral gyrus medial segment, post central gyrus, opercular part of inferior frontal gyrus, triangular part of inferior frontal gyrus (pars triangularis), frontal pole, gyrus rectus, lateral orbital gyrus, precentral gyrus medial segment, precentral gyrus, superior frontal gyrus, supramarginal gyrus, medial frontal cortex, middle frontal gyrus, middle frontal gyrus, superior frontal gyrus medial segment, supplementary motor area; and (2) expanded cortical composite: extended frontal composite plus posterior cingulate cortex, inferior parietal lobule, superior temporal gyrus, anterior cingulate cortex, superior parietal lobule, middle temporal gyrus, precuneus cortex, paracentral lobule.

### Statistics

According to the protocol, the planned cohort B sample size of 240 allows a minimal detectable effect size of 0.38 (Cohen’s *D*) with two-sided alpha of 0.05, 80% power and 10% dropout. Power calculations and endpoint selection were based on a pilot study (cohort A) of 36 patients with HD (15 EM and 21 LP) treated with pepinemab or placebo in a double-blinded comparison for 6 months. The 2CARE study provided additional information on estimates of variance at month 18 (ref. ^67^).

The statistical analysis protocol, finalized before locking the database and unblinding, specified a plan to control the overall type I error rate in multiple testing of two coprimary analyses plus a series of secondary analyses to be tested in hierarchy. The coprimary endpoints were the two-item HD–CAB family (OTS and PTAP) and CGIC. Both coprimary endpoints were required to meet a critical one-sided *P* value of 0.025 (equivalent to two-sided alpha of 0.05) for a successful trial overall. The success of the two-item HD–CAB family was assessed according to the Hochberg procedure for multiple testing between two items. Because the coprimary endpoints did not collectively reach the threshold needed to declare a successful finding, the prespecified hierarchy of secondary endpoints was not tested formally. Thus, stated *P* values for all statistical tests besides the coprimary efficacy analyses were not corrected for multiplicity and are thus presented as nominal and not under alpha control.

The efficacy analyses for this study were performed in a mITT population. Missing values were not imputed before fitting the models for statistical testing, and estimation and inference were based on estimates of the difference or ratio between means (that is, PEPI versus PBO) at month 17. No data beyond month 17 were included in the efficacy analyses.

For continuous outcomes, the dependent variable is generally change from baseline over time, and these values were analyzed using MMRM with categorical time, treatment group, screening value (if applicable) and time by treatment as explanatory variables and with an unstructured covariance structure. By contrast, CGIC is inherently a change score and does not have a baseline value for inclusion in the model. The relevant summary statistics presented for continuous outcomes include LS means, standard errors, 95% CI and the *P* value from an MMRM. For binary outcomes, the results presented include proportions by arm, an odds ratio, exact CIs and *P* values from Fisher’s exact test. The *P* values presented are generally one-sided to reflect the known direction of benefit; two-sided *P* values are presented only for exploratory outcomes where the direction of benefit was not necessarily established. Statistical analyses of clinical data were performed using SAS software v.9.4.

### PK/PD

Blood samples for the analysis of pepinemab drug levels were obtained on a monthly basis through to the last visit. Blood was collected on all dosing days before the first and after the last infusion. Blood draw for SEMA4D saturation in whole blood, T cell SEMA4D levels, total soluble SEMA4D levels in serum and exploratory serum cytokine and biomarker analysis was collected at baseline, visits 1 and 3 and every 3 months thereafter. In a subset of subjects who volunteered (*n* = 54), CSF was collected by lumbar puncture during one visit among visits 13–18 (mean, 15.6; median, 15.0), within 24 h post infusion, to be evaluated for pepinemab concentration, total SEMA4D and other biomarkers. Validated assays were utilized. Briefly, pepinemab drug levels were measured using a sandwich ELISA format with an anti-idiotype antibody, and total soluble SEMA4D was measured using a sandwich ELISA format with two SEMA4D-specific monoclonal antibodies that do not react with the same epitope of pepinemab, thus allowing measurement of both free and bound soluble SEMA4D^18,19^.

### Ethics

Institutional review board (IRB) approvals for the study protocol, amendments and informed consent documents were obtained before use in the study; written informed consent was obtained from study participants before the initiation of study procedures. This study utilized both a central IRB—Western Institutional Review Board—as well as local IRBs at sites that did not utilize the central IRB. As per the International Classification of Functioning, Disability and Health, participants received US$450 for each routine visit, an additional US$250 for LP/CSF and an additional US$250 for radioligands targeting translocator protein (TSPO)-PET; an additional reimbursement for transportation or lodging costs was considered on a case-by-case basis. This study was conducted in accordance with the Declaration of Helsinki, International Conference on Harmonization guidelines and applicable portions of the United States Code of Federal Regulations. The ClinicalTrials.gov identifier (no. NCT02481674) was obtained before study initiation. All experiments including human specimens were performed in compliance with the relevant ethical regulations.

### Reporting summary

Further information on research design is available in the Nature Research Reporting Summary linked to this article.

## Online content

Any methods, additional references, Nature Research reporting summaries, source data, extended data, supplementary information, acknowledgements, peer review information; details of author contributions and competing interests; and statements of data and code availability are available at 10.1038/s41591-022-01919-8.

### Supplementary information

Supplementary Information Supplementary Figs. 1 and 2 and Table 1.

Reporting Summary

## Figures and Tables

**Extended Data Table 1:**
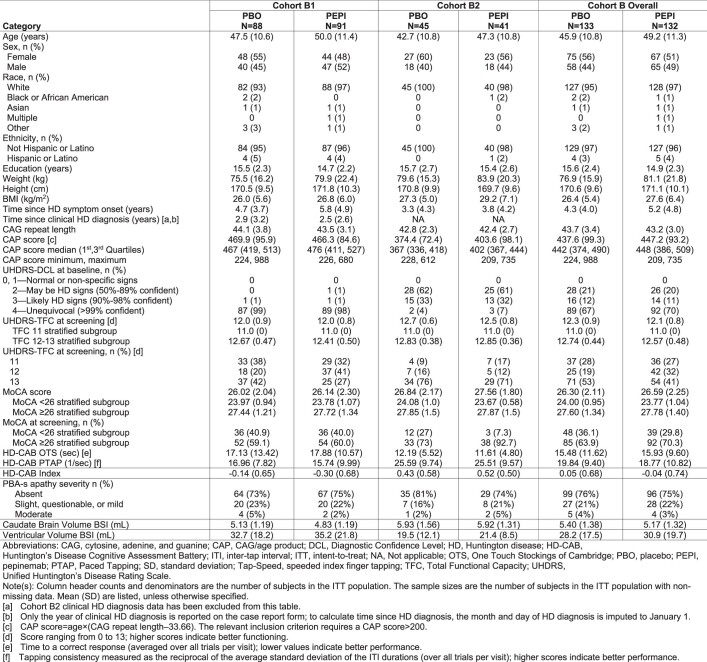
Demographics and baseline characteristics for all cohorts (ITT population)

**Extended Data Table 2:**
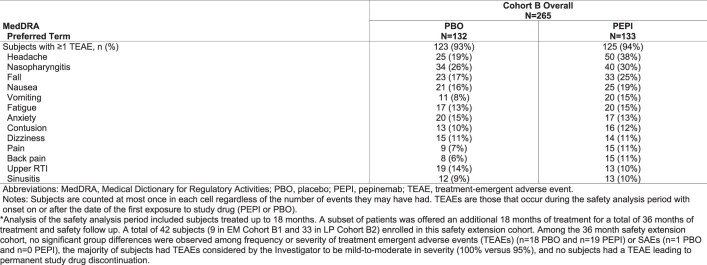
Common TEAEs (≥10% of Subjects) Cohort B overall for the safety analysis period*

**Extended Data Table 3:**
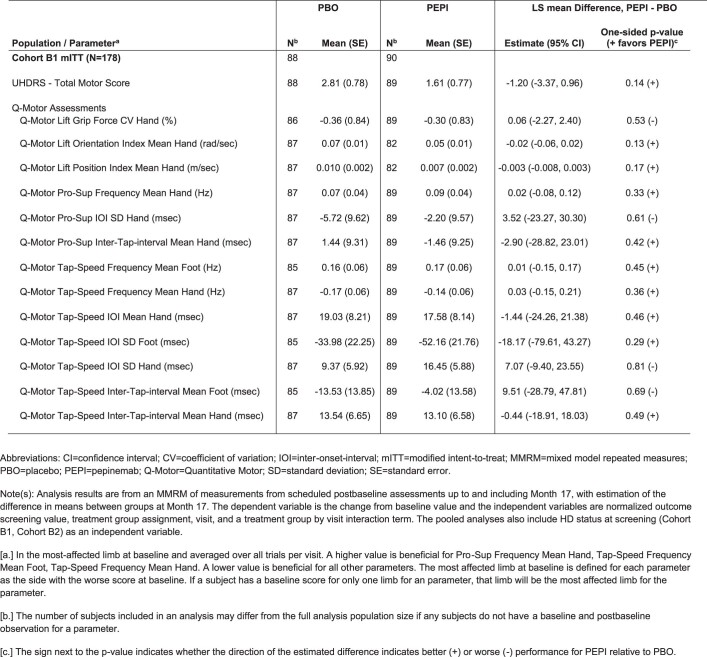
Exploratory analysis of motor assessments in EM Cohort B1, mean change from Baseline to Month 17

**Extended Data Table 4:**
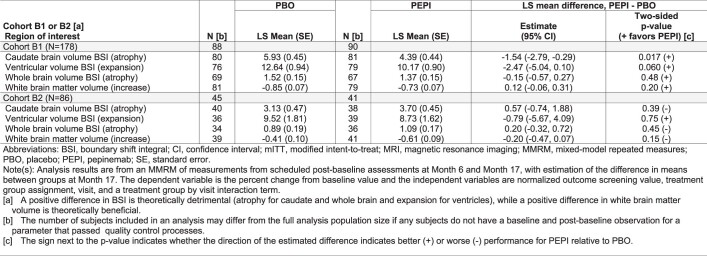
Exploratory analyses of volumetric MRI in cortical brain regions of interest in EM Cohort B1 and LP Cohort B2, percent change from Baseline to Month 17

**Extended Data Table 5:**
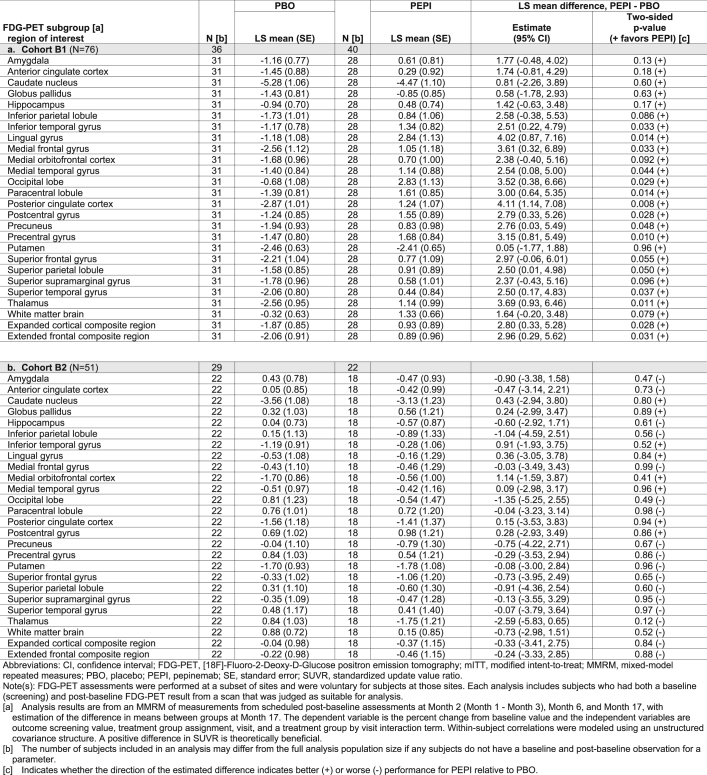
Exploratory analysis of FDG-PET SUVR in brain regions of interest in EM Cohort B1 and LP Cohort B2, percent change from Baseline to Month 17 **a**. EM Cohort B1. **b**. LP Cohort B2

**Extended Data Table 6:**
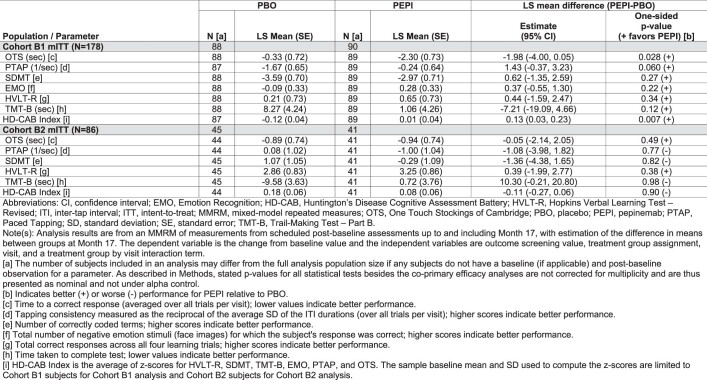
Exploratory analyses of HD-CAB Index and six components in EM Cohort B1 and LP Cohort B2 (mITT Population), mean change from Baseline to Month 17

**Extended Data Table 7:**
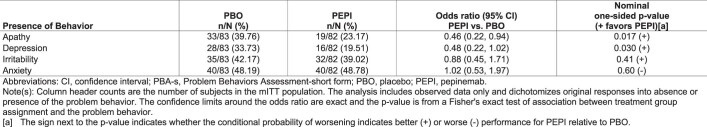
Post-hoc analyses of selected PBA-s severity subscores at Month 17 in EM Cohort B1

## Data Availability

Requests for access to the patient-level data from this study can be submitted via email to medinfo@vaccinex.com with detailed proposals for use of information. A signed data access agreement with the sponsor is required before accessing shared data. All requests for study protocol and data will be reviewed by the study sponsor, Vaccinex, to verify whether the request is subject to any intellectual property or confidentiality obligations. Patient-related data were generated as part of a clinical trial and may be subject to patient confidentiality. The SIGNAL study is registered with ClinicalTrials.gov (no. NCT02481674).
