# Publisher Correction: Pepinemab antibody blockade of SEMA4D in early Huntington’s disease: a randomized, placebo-controlled, phase 2 trial

**DOI:** 10.1038/s41591-022-02070-0

**Published:** 2022-10-04

**Authors:** Andrew Feigin, Elizabeth E. Evans, Terrence L. Fisher, John E. Leonard, Ernest S. Smith, Alisha Reader, Vikas Mishra, Richard Manber, Kimberly A. Walters, Lisa Kowarski, David Oakes, Eric Siemers, Karl D. Kieburtz, Maurice Zauderer, the Huntington Study Group SIGNAL investigators, Elise Kayson, Jody Goldstein, Richard Barbano, Karen Marder, Praveen Dayalu, Herminia Diana Rosas, Sandra Kostyk, John Kamholz, Brad Racette, Jee Bang, Daniel Claassen, Katherine McDonell, Stewart Factor, Francis Walker, Clarisse Goas, Joanne Wojcieszek, Lynn A. Raymond, Jody Corey-Bloom, Victor Sung, Marissa Dean, Michael Geshwind, Alexandra Nelson, Samuel Frank, Kathrin LaFaver, Andrew Duker, Lawrence Elmer, Ali Samii, Yi-Han Lin, Sylvain Chouinard, Lauren Seeberger, Burton Scott, James Boyd, Nikolaus McFarland, Erin Furr Stimming, Oksana Suchowersky, Claudia Testa, Karen Anderson

**Affiliations:** 1 New York University Langone Health and The Marlene and Paolo Fresco Institute for Parkinson’s and Movement Disorders, New York, NY USA; 2 Vaccinex, Inc., Research, Rochester, NY USA; 3 IXICO, London, UK; 4 WCG Statistics Collaborative, Inc., Washington, DC USA; 5 University of Rochester Medical Center, Rochester, NY USA; 6 Siemers Integration LLC, Zionsville, IN USA; 7 Clinical Trials Coordination Center, University of Rochester, Rochester, NY USA; 8 Columbia University, New York, NY USA; 9 University of Michigan, Ann Arbor, MI USA; 10 Massachusetts General Hospital, Boston, MA USA; 11 Ohio State University, Columbus, OH USA; 12 University of Iowa, Iowa City, IA USA; 13 Washington University, St. Louis, MO USA; 14 Johns Hopkins University, Baltimore, MD USA; 15 Vanderbuilt University, Nashville, TN USA; 16 Emory University, Atlanta, GA USA; 17 Wake Forest University, Winston-Salem, NC USA; 18 Indiana University, Bloomington, IN USA; 19 University of British Columbia, Vancouver, British Columbia Canada; 20 UCSD, San Diego, CA USA; 21 University of Alabama at Birmingham, Birmingham, AL USA; 22 UCSF, San Francisco, CA USA; 23 Beth Israel Deaconess Medical Center, Boston, MA USA; 24 University of Louisville, Louisville, KY USA; 25 University of Cincinnati, Cincinnati, OH USA; 26 University of Toledo, Toledo, OH USA; 27 University of Washington, Seattle, WA USA; 28 CHUM, Montreal, Quebec Canada; 29 University of Colorado, Aurora, CO USA; 30 Duke University, Durham, NC USA; 31 University of Vermont, South Burlington, VT USA; 32 University of Florida Gainesville, Gainesville, FL USA; 33 University of Texas Houston, Houston, TX USA; 34 University of Alberta, Edmonton, Alberta Canada; 35 University of North Carolina at Chapel Hill, Chapel Hill, NC USA; 36 Georgetown University, Washington, DC USA

**Keywords:** Huntington's disease, Drug development

Correction to: *Nature Medicine* 10.1038/s41591-022-01919-8, published online 8 August 2022.

In the version of this article initially published, in the first and final paragraphs of the Results section, the dosing schedule for pepinemab administration was described in error as “four times per week” instead of “every four weeks,” while in the Fig. 4b color key, the bottom, dark green key now reading “Much improved” appeared originally as “Minimally improved.” The errors have been corrected in the HTML and PDF versions of the article.

