# Supplementary material for: Pepinemab antibody blockade of SEMA4D in early Huntington’s disease: a randomized, placebo-controlled, phase 2 trial
Source: Nat Med. 2022 Aug 8;28(10):2183–93. doi: 10.1038/s41591-022-01919-8 (PMC9361919; doi:10.1038/s41591-022-01919-8)
Supplement: Supplementary file 1 — Supplementary Figs. 1 and 2 and Table 1. [file 41591_2022_1919_MOESM1_ESM.pdf]

nature portfolio

https://doi.org/10.1038/s41591-022-01919-8

Supplementary information

Pepinemab antibody blockade of SEMA4D in early Huntington's disease: a randomized, placebo-controlled, phase 2 trial

In the format provided by the authors and unedited

Supplementary Information

Pepinemab antibody blockade of SEMA4D in early Huntington's Disease: a randomized, placebo controlled, phase 2 trial

Andrew Feigin[1], Elizabeth E. Evans[2], Terrence L. Fisher[2], John E. Leonard[2], Ernest S. Smith[2], Alisha Reader[2], Vikas Mishra[2], Richard Manber[3], Kimberly A. Walters[4], Lisa Kowarski[4], David Oakes[5], Eric Siemers[6], Karl D. Kieburtz[5], Maurice Zauderer[2], and the Huntington Study Group SIGNAL investigators[*]

Corresponding author: Dr. Maurice Zauderer, Vaccinex, Inc., 1895 Mt. Hope Avenue, Rochester, NY, USA; mzauderer@vaccinex.com; (585) 503-5949.

[1]New York University Langone Health, and The Marlene and Paolo Fresco Institute for Parkinson's and Movement Disorders, New York, New York, USA. [2]Vaccinex, Inc., Research, Rochester, New York, USA. [3]IXICO, London, England. [4]WCG Statistics Collaborative, Inc., Washington DC, USA. [5]University of Rochester Medical Center, Rochester, New York, USA. [6]Siemers Integration LLC, Zionsville, Indiana, USA. *A list of authors and their affiliations appears at the end of the paper.

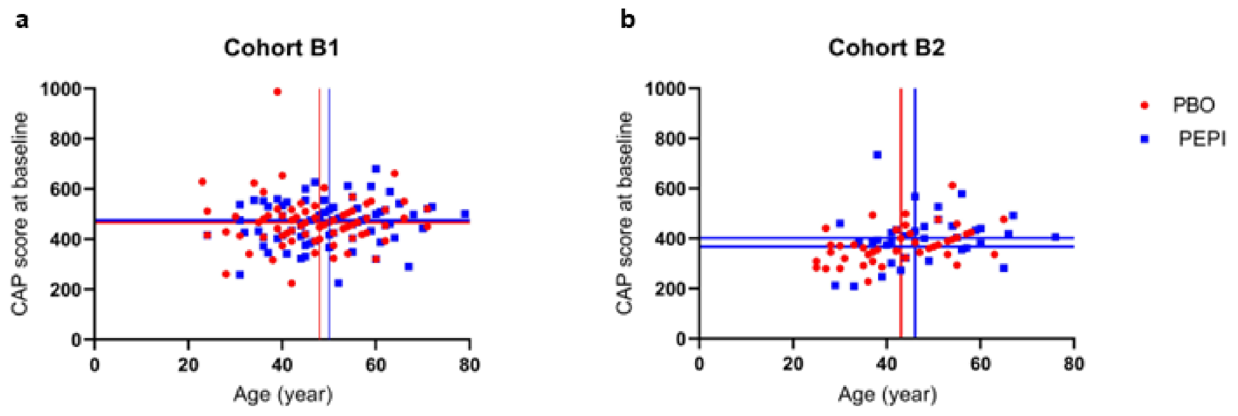

Supplementary Fig. 1: Baseline CAP scores and age. a. Cohort B1**. b.** Cohort B2. The symbols represent pepinemab (PEPI) in blue and placebo (PBO) in red; lines represent median.

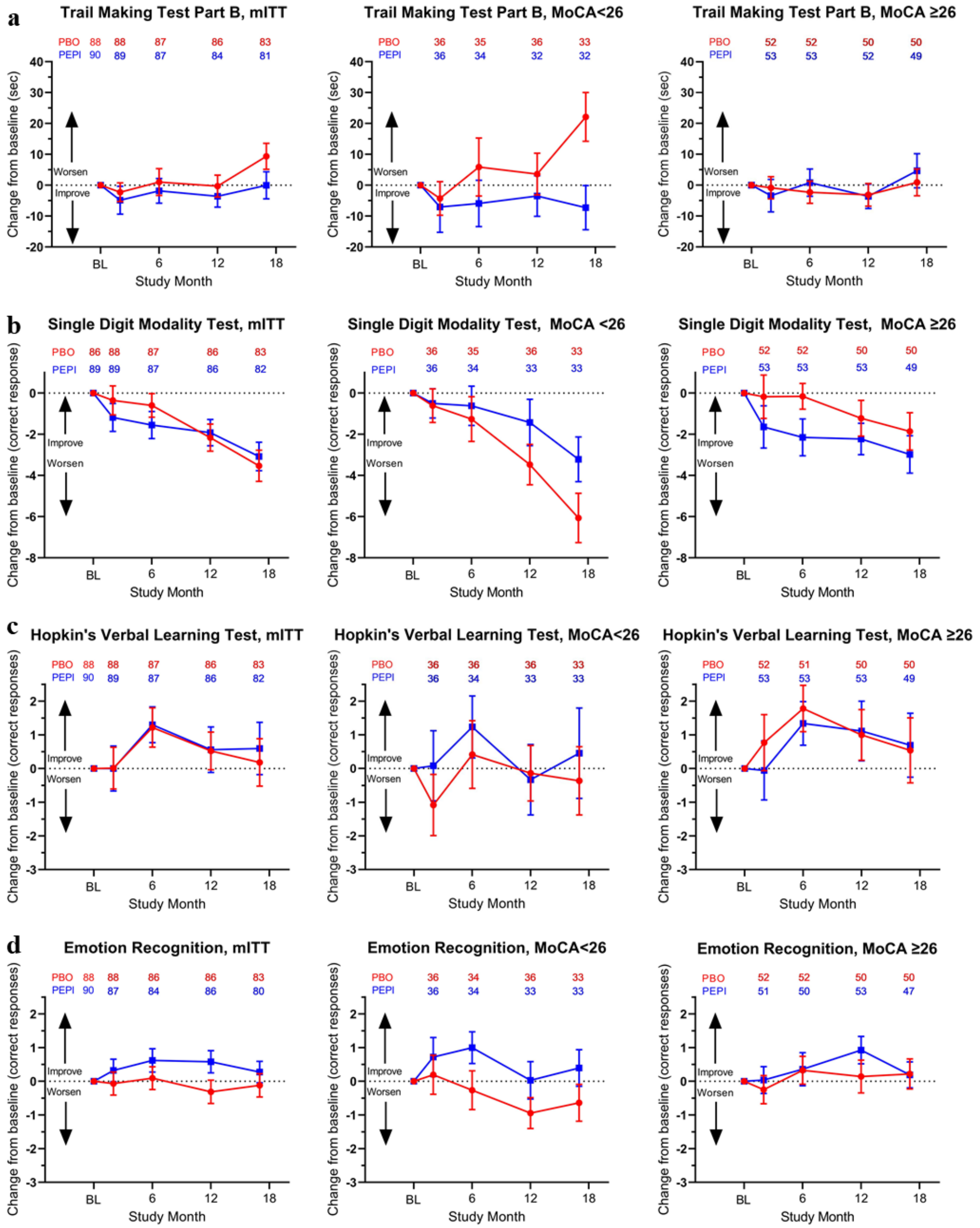

Supplementary Fig. 2: Post-hoc subgroup analysis of baseline MoCA as a biomarker for treatment response in early HD. Observed mean changes from baseline by treatment group over time for the EM Cohort B1 stratified by baseline MoCA scores of <26, or 26-30, considered to be normal cognitive function. Assessments of a. Trail making test - part B, b. Single digit modality test, c. Hopkins verbal learning test - revised, and d. Emotion recognition. The error bars in each panel show one standard error on either side of the mean, and the sample sizes at each timepoint for each group are listed above the profile lines. The symbols represent PEPI (blue) and PBO (red).

Supplementary Table 1: List of study coordinators, medical monitor, and safety monitoring committee

Study coordinators	Candace Cromer & Jenna Smith	University of Alabama at Birmingham, Birmingham, AL
	Chase Snell & Jordan Castleton	UCSD, San Diego, CA
	Julia Glueck	UCSF, San Francisco, CA
	Erin Clark & Mary Cook	University of Colorado, Aurora, CO
	Hope Heller, Erin Koppel, & Robin Kuprewicz	Georgetown University, Washington DC
	Kyle Rizer	University of Florida Gainesville, Gainesville, FL
	Elaine Sperin	Emory University, Atlanta, GA
	Owen Wade	University of Iowa, Iowa City, IA
	Andrea Hurt	Indiana University, Bloomington, IN
	Annette Robinson	University of Louisville, Louisville, KY
	Jacqueline Fung, Noah Allanoff, & Wilanda Gabriel	Beth Israel Deaconess Medical Center, Boston, MA
	Courtney Jordan & Natilie Conners	Massachusetts General Hospital, Boston, MA
	Mollie Jenkes	John's Hopkins University, Baltimore, MD
	Angela Stovall & Michael Hadden	University of Michigan, Ann Arbor, MI
	Melissa Ammel	Washington University, St. Louis, MO
	Kate Beck & Lisa Gauger	Duke University, Durham, NC
	Summer Harris	Wake Forest Universtiy, Winston-Salem, NC
	Amy Chesire	University of Rochester, Rochester, NY
	Paula Wasserman	Columbia University, New York, NY
	Brandi Crews	University of Cincinnati, Cincinnati, OH
	Allison Daley & Katherine Ambrogi	Ohio State University, Columbus, OH
	Kristen Schuchmann, Julia Spears, Nancy Vollmar, & Stephanie Wilson	University of Toledo, Toledo, OH
	Danielle Buchanan	Vanderbilt University, Nashville, TN
	Brittany Duncan & Jamie Sims	University of Texas Houston, Houston, TX
	Kelly Huckstep, Heather Ward, & Ginger Norris	Virginia Commonwealth University, Richmond, VA
	Emily Houston	University of Vermont, South Burlington, VT
	Debra Del Castillo	University of Washington, Seattle, WA
	Paul McCann	University of Alberta, Edmonton, AL
	Mike Adurogbangba & Joji Decolongon	University of British Columbia, Vancouver, BC
	Martine Comeau & Marie Martin	CHUM, Montreal, QB
Medical Monitor	Martin Niethammer	North Shore University Hospital, Great Neck, NY
Safety Monitoring Committee	Michael McDermott	University of Rochester, Rochester, NY
	Timothy Greenamyre	University of Pittsburgh, Pittsburgh, PA
	Donald Higgins	Samuel S. Stratton VA Medical Center, Albany, NY
