# Supplementary material for: Pepinemab antibody blockade of SEMA4D in early Huntington’s disease: a randomized, placebo-controlled, phase 2 trial
Source: Nat Med. 2022 Aug 8;28(10):2183–93. doi: 10.1038/s41591-022-01919-8 (PMC9361919; doi:10.1038/s41591-022-01919-8)
Supplement: Supplementary file 2 — Reporting Summary [file 41591_2022_1919_MOESM2_ESM.pdf]

nature portfolio

Corresponding author(s): Maurice Zauderer

Last updated by author(s): Jun 13, 2022

Reporting Summary

Nature Portfolio wishes to improve the reproducibility of the work that we publish. This form provides structure for consistency and transparency in reporting. For further information on Nature Portfolio policies, see our Editorial Policies and the Editorial Policy Checklist.

Statistics

For all statistical analyses, confirm that the following items are present in the figure legend, table legend, main text, or Methods section.

n/a	Confirmed	
☐	☒	The exact sample size (*n*) for each experimental group/condition, given as a discrete number and unit of measurement
☐	☒	A statement on whether measurements were taken from distinct samples or whether the same sample was measured repeatedly
☐	☒	The statistical test(s) used AND whether they are one- or two-sided<br>*Only common tests should be described solely by name; describe more complex techniques in the Methods section.*
☐	☒	A description of all covariates tested
☐	☒	A description of any assumptions or corrections, such as tests of normality and adjustment for multiple comparisons
☐	☒	A full description of the statistical parameters including central tendency (e.g. means) or other basic estimates (e.g. regression coefficient) AND variation (e.g. standard deviation) or associated estimates of uncertainty (e.g. confidence intervals)
☐	☒	For null hypothesis testing, the test statistic (e.g. *F*, *t*, *r*) with confidence intervals, effect sizes, degrees of freedom and *P* value noted<br>*Give P values as exact values whenever suitable.*
☒	☐	For Bayesian analysis, information on the choice of priors and Markov chain Monte Carlo settings
☐	☒	For hierarchical and complex designs, identification of the appropriate level for tests and full reporting of outcomes
☐	☒	Estimates of effect sizes (e.g. Cohen's *d*, Pearson's *r*), indicating how they were calculated

Our web collection on statistics for biologists contains articles on many of the points above.

Software and code

Policy information about availability of computer code

Data collection	Omnicomm Inc. eClinical Solutions Version 5.2 was utilized for data collection on SIGNAL.
Data analysis	Statistical analyses of clinical data were performed using SAS software, version 9.4. MRI pre-processing software included MRIcron DICOM to NIfTI conversion (version 6/2013), DCMTK extraction of orientation information (version of 3.5.4), Image registration toolkit (IRTK, version 1.95) for mulit-atlas brain extraction, registrations, N4BiasFieldCorrection (version 1.9 from ANTs package), MIDAS for semi-automated delineation (version 5.11.1), KN-BSI for boundary shift integral calculation of volume change (version 1.2), NiftyReg/NiftiSeg (version 0.9.4), NifTK for BSI computation (version 12.11).

For manuscripts utilizing custom algorithms or software that are central to the research but not yet described in published literature, software must be made available to editors and reviewers. We strongly encourage code deposition in a community repository (e.g. GitHub). See the Nature Portfolio guidelines for submitting code & software for further information.

Data

Policy information about availability of data

All manuscripts must include a data availability statement. This statement should provide the following information, where applicable:

- Accession codes, unique identifiers, or web links for publicly available datasets
- A description of any restrictions on data availability
- For clinical datasets or third party data, please ensure that the statement adheres to our policy

All requests for study protocol and data will be reviewed by the study sponsor, Vaccinex, to verify whether the request is subject to any intellectual property or confidentiality obligations. Patient-related data were generated as part of a clinical trial and may be subject to patient confidentiality. Requests for access to the patient-level data from this study can be submitted via email to medinfo@vaccinex.com with detailed proposals for use of information. A signed data access

agreement with the sponsor is required before accessing shared data. The data that support the findings of this study are available from the corresponding author upon reasonable request. The SIGNAL study is registered with ClinicalTrials.gov, number NCT02481674.

Field-specific reporting

Please select the one below that is the best fit for your research. If you are not sure, read the appropriate sections before making your selection.

☒ Life sciences ☐ Behavioural & social sciences ☐ Ecological, evolutionary & environmental sciences

For a reference copy of the document with all sections, see nature.com/documents/nr-reporting-summary-flat.pdf

Life sciences study design

All studies must disclose on these points even when the disclosure is negative.

Sample size	According to the protocol, the planned Cohort B sample size of 240 allows a minimal detectable effect size of 0.38 (Cohen's D) with two-sided alpha of 0.05, 80% power, and 10% dropout. Power calculations and endpoint selection were based on an earlier pilot study (Cohort A) of 36 HD patients (15 Early Manifest and 21 Late Prodromal) treated with pepinemab or placebo in a double-blinded comparison for six months. The 2CARE study provided additional information ons estimates of variance at Month 18.
Data exclusions	No individual data was excluded from reported endpoints.
Replication	Group sizes were sufficiently large to afford multiple subject replicates for each assessment and time point. In addition, one previous pilot study was conducted and trends of safety, tolerability, PK, cognitive and FDG-PET imaging results were reproduced in current report.
Randomization	Subjects who satisfied all eligibility criteria were participants and were randomized to 1 of 2 treatment arms through an Interactive Web Response System (IWRS). Subjects in Cohorts B1 and B2 were independently randomized. This system also prescribed for each patient specific numbered vials of pepinemab or matching placebo. The placebo used in the study matched the vialed pepinemab in that the vial, vial stopper and overseal, formulation, vial fill volume and label appearance were identical to that of the vialed pepinemab.
Blinding	The subjects, site investigators, site personnel, study statisticians, as well as representatives of these organizations and staff at Vaccinex were blinded as to treatment assignments until database lock. The investigational agent and placebo were in vials identical in appearance. During the course of the study, the Safety Monitoring Committee (SMC) maintained access to treatment code information.

Reporting for specific materials, systems and methods

We require information from authors about some types of materials, experimental systems and methods used in many studies. Here, indicate whether each material, system or method listed is relevant to your study. If you are not sure if a list item applies to your research, read the appropriate section before selecting a response.

Materials & experimental systems

n/a	Involved in the study	
☐	☒	Antibodies
☒	☐	Eukaryotic cell lines
☒	☐	Palaeontology and archaeology
☒	☐	Animals and other organisms
☐	☒	Human research participants
☐	☒	Clinical data
☒	☐	Dual use research of concern

Methods

n/a	Involved in the study	
☒	☐	ChIP-seq
☒	☐	Flow cytometry
☐	☒	MRI-based neuroimaging

Antibodies

Antibodies used	Pepinemab (VX15/2503) is a humanized IgG4 monoclonal antibody with a hinge mutation to prevent in vivo Fab arm recombination. Bulk pepinemab (Catalent Pharma Solutions, Madison, WI) was produced using a proprietary CHO cell line.
Validation	The bulk antibody was purified using standard techniques and formulated at approximately 20 mg/mL in preservative-free 20 mM sodium acetate, pH 5.4, containing 130 mM sodium chloride and 0.02 % polysorbate 80. Pepinemab and matching placebo were supplied by the Sponsor as single-use vials.

Human research participants

Policy information about studies involving human research participants

Population characteristics	See Extended Data Table 2.

Recruitment	Eligible participants were aged 21 years and older, had genetically confirmed presence of ≥36 cytosine-adenine-guanine (CAG) repeats in one huntingtin gene and no features of juvenile HD (Westphal variant). EM participants were defined by UHDRS-TFC ≥11; they were determined by the site investigator to have a clinical diagnosis of HD as defined by a DCL of 4. LP was defined as DCL of 2 or 3 and a CAP score of >200. CAP score, a measure of HD mutation burden, was calculated as the product of age x (CAG-33.66) 55. Stable dosages of concomitant medications (including tetrabenazine and deutetrabenazine) were permitted if initiated at least one month prior to baseline (Visit 0), with the exception of newly prescribed anxiolytics for the use of premedication prior to imaging at screening, which were permitted on a case-by-case basis. Exclusion criteria include participation in an investigational drug or device study within 30 days of baseline, or 180 days if previous investigational drug was a monoclonal antibody, therapeutic, suicide risk, MoCA score ≤22, ECG abnormalities at screening, pregnancy, conditions which would exclude MRI participation. Per the ICF, participants received $450 for each routine visit, an additional $250 for LP/CSF, and additional $250 for TSPO-PET, and additional reimbursement for transportation or lodging costs was be considered on a case-by-case basis.
Ethics oversight	Institutional review board approvals for the study protocol, amendments, and informed consent documents were obtained before use in the study; written informed consent was obtained from study participants before the initiation of study procedures. This study was conducted in accordance with the Declaration of Helsinki, International Conference on Harmonization (ICH) guidelines, and applicable portions of the United States Code of Federal Regulations. The ClinicalTrials.gov identifier (NCT02481674) was obtained before study initiation. All experiments including human specimens were performed in compliance with the relevant ethical regulations. This study utilized both a central IRB; Western Institutional Review Board (WIRB) as well as local IRBs at sites that did not utilize the central IRB.

Note that full information on the approval of the study protocol must also be provided in the manuscript.

Clinical data

Policy information about clinical studies

All manuscripts should comply with the ICMJE guidelines for publication of clinical research and a completed CONSORT checklist must be included with all submissions.

Clinical trial registration	NCT02481674
Study protocol	Clinicaltrials.gov
Data collection	SIGNAL Cohort B is a Phase 2, multi-center, randomized (1:1), double-blind, placebo controlled, parallel-group study of pepinemab in subjects with EM (Cohort B1) and LP (Cohort B2) HD (https://clinicaltrials.gov/ct2/show/NCT02481674) (VX15-2503-N-131B). A total of 265 subjects (179 EM, and 86 LP) were enrolled at 30 outpatient clinical sites in USA and Canada. Subjects were enrolled from 28 December 2015 through 31 December 2018. Data was collected at each clinical site and entered into the EDC (eClinical Solutions), which was reviewed regularly by the CRAs. The EDC was centrally managed and reviewed by the clinical CRO.
Outcomes	Tolerability, defined as the ability to complete the study on the assigned study arm, accounted for subject's study disposition (e.g. reason for study discontinuation of "Did not tolerate study drug"), treatment disposition, and duration of exposure. Adverse events (AEs) were monitored monthly on each subject during the study period, defined as from signing informed consent through the final study contact. A treatment-emergent AE (TEAE) is defined as an adverse event with onset on or after the date of first dose of study drug. AEs are coded using the Medical Dictionary for Regulatory Activities (MedDRA) version 16.1. The Investigator assessed the causality of each AE to study drug and the severity of each AE using his/her clinical expertise and designated a grade to each AE per the current Common Terminology Criteria for Adverse Events (CTCAE). Columbia-Suicide Severity Rating Scale and Hospital Anxiety and Depression Scale were assessed. The Huntington's Disease Cognitive Assessment Battery (HD-CAB) was measured at six visits during the Primary Analysis Period: screening, baseline, and Months 2, 6, 12, and 17. The HD-CAB is comprised of six component tests two of which are PTAP and OTS, and are described in METHODS. CGIC is a single item questionnaire that asks the investigator to assess a subject's HD symptoms compared to immediately before starting study drug, using a 7-point Likert Scale, ranging from very much worse (-3) to very much improved (+3), to assess overall response to study drug relative to baseline. CGIC was evaluated at four visits during the Primary Analysis Period: Months 3, 5, 11, and 17. The Total Functional Capacity (TFC) scale is a component of the Unified Huntington's Disease Rating Scale (UHDRS; together referred to as "UHDRS-TFC") and has been used in premanifest and manifest HD populations in many observational studies and randomized controlled trials. The UHDRS-TFC score, is the sum of five items, ranges from 0 to 13, with a higher score representing better functioning. Q-motor and TMS allow objective monitoring of unintentional motor side-effects. The UHDRS-TMS assesses motor features of HD with standardized ratings in the following five domains: eye movement, chorea (jerky movement), dystonia (muscle spasm and twisting), bradykinesia (slowness in movement), and rigidity (stiffness). Items in each of the five domains are individually rated on a five-point scale ranging from 0 (normal) to 4 (most severe impairment). The sum of the scores of all 31 items is referred to as the UHDRS-TMS. The range of the UHDRS-TMS is 0 to 124, with higher scores indicating more severe motor impairment. (REF) The Q-Motor battery is composed of pre-calibrated and temperature-controlled force transducers and 3D position sensors that are used to assess (1) grasping forces, (2) involuntary choreatic movements, (3) regularity of index finger tapping, and (4) regularity of alternating pronation/supination hand movements. PBA-s is a semi structured clinical interview that contains 11 items, each measuring a different behavioral problem which is rated for both severity and frequency on a five-point scale (0–4).

Magnetic resonance imaging

Experimental design

Design type	Volumetric MRI only
Design specifications	NA; The design type question above is about block or event-based design, which refers to functional MRI, but we are only doing volumetric MRI here, so there is no design spec.

Behavioral performance measures | NA; no behavioral measures taken as acquisiton was resting state volumetric scans only.

Acquisition

Imaging type(s): Structural, T1W sequence

Field strength: 3T

Sequence & imaging parameters:
3DT1W Sequence image parameters: Gradient echo T1W sequence, Cartesian readout, based on ADNI 1 guidelines.
Voxel size: 1x1x1mm, FOV: 256mm
Sequence type: FFE (Philips), Bravo (GE), TFL (Siemens)
Orientation: Sagittal
TE/TR/flip angle: vary between scanners/sequences, but typical example:
Siemens: TE 3.3ms, TR 2530ms, FA 7 deg, TI 1100ms

Area of acquisition: Whole brain

Diffusion MRI ☐ Used ☒ Not used

Preprocessing

Preprocessing software: MRIcron DICOM to NIfTI conversion (version 6/2013), DCMTK extraction of orientation information (version of 3.5.4), Image registration toolkit (IRTK, version 1.95) for mulit-atlas brain extraction, registrations, N4BiasFieldCorrection (version 1.9 from ANTs package), MIDAS for semi-automated delineation (version 5.11.1), KN-BSI for boundary shift integral calculation of volume change (version 1.2), NiftyReg/NiftiSeg (version 0.9.4), NifTK for BSI computation (version 12.11), LEAP (Learning Embeddings for Atlas Propagation) and LLEAP (Longitudinal LEAP) used for regional brain segmentations.

Normalization: Individual brain volume measurements can be adjusted for intracranial volume to correct for subject-variability in head size. Following the work of (Buckner 2004), an affine scaling factor is used, which relates the subject's brain size to that of an atlas to calculate intracranial volume (ICV). This measurement of ICV is referred to as pseudo total intracranial volume (pTIV), and entails affinely registering the patient T1-weighted image to an atlas and calculating the overall scaling factor as the magnitude of the scaling vector in the transformation matrix calculated from the registration. Rigid and affine registrations are used with the MNI atlas.

Normalization template: Subject 3DT1w image affine transformation to whole head MNI atlas in MNI space, with MNI305 template

Noise and artifact removal: N4 bias field correction

Volume censoring: NA; Each MRI acquisition was a 3D single image, rather than a 4D time series, so no volume censoring is required.

Statistical modeling & inference

Model type and settings: All sites and their respective 3T MRI scanners were qualified prior to the start of the study. The protocol included the following scan sequences; 1. Localizer, 2. T1w, 3. Field Map DTI, 4. DWI, 5. T2_SPACE, 6. T2_FLAIR, 7. Field Map fMRI, 8. Resting State fMRI, 9. PD, but analysis only involved analysis of volumetric changes (T1w). IXICO reports the pseudo total intracranial volume (pTIV) factor, which is a measure of how a subject's head size compares to the standard template. Screening results were normalized to the pTIV to account for differences in head size. The normalized screening result for each brain region was calculated as screening volume / pTIV factor. At each post-baseline timepoint, percentage change from baseline was calculated as (reported change value / reported screening value) × 100.

Effect(s) tested: Volumetric change from baseline

Specify type of analysis: ☐ Whole brain ☒ ROI-based ☐ Both

Anatomical location(s): automated labeling algorithms

Statistic type for inference (See Eklund et al. 2016): voxel-wise

Correction: NA; Imaging vendor reported absolute and and % volume change, with no correction needed.

Models & analysis

n/a	Involved in the study
☒	☐ Functional and/or effective connectivity
☒	☐ Graph analysis
☐	☒ Multivariate modeling or predictive analysis

Multivariate modeling and predictive analysis: The statistical analysis plan, finalized prior to locking the database and unblinding, specified a plan to control the overall Type I error rate in multiple testing of two co-primary analyses plus a series of secondary analyses to be tested in hierarchy. The co-primary endpoints: were the two-item HD-CAB family (OTS and PTAP) and

the CGIC. Both co-primary endpoints were required to meet a critical one-sided p-value of 0.025 (equivalent to a two-sided alpha of 0.05) for a successful trial overall. The success of the HD-CAB family was assessed according to the Hochberg procedure for multiple testing among the two items. Since the co-primary endpoints did not collectively reach the threshold needed to declare a successful finding, the prespecified hierarchy of secondary endpoints were not tested formally. Thus, stated p-values for all statistical tests besides the co-primary efficacy analyses are not corrected for multiplicity and are thus presented as nominal and not under alpha control.
For the continuous outcomes, the dependent variable is generally change from baseline over time, and these values were analyzed using mixed models for repeated measures (MMRM) with categorical time, treatment group, screening value (if applicable), and time by treatment as explanatory variables and with an unstructured covariance structure. Alternately, CGIC is inherently a change score and does not have a baseline value to include in the model. The relevant summary statistics presented for continuous outcomes include the LS means, standard errors (SE), 95% confidence intervals (CI), and the p-value from an MMRM.
For the binary outcomes, the results presented include proportions by arm, an odds ratio, exact confidence intervals, and the p-value from a Fisher's exact test. The p-values presented are generally one-sided to reflect the known direction of benefit; two-sided p-values are presented only for exploratory outcomes where the direction of benefit is not necessarily established.
